# Supplementary figures and images for: MIAAIM: Multi-omics image integration with dimensional reduction for tissue state mapping
Source: PLoS Comput Biol. 2026 May 26;22(5):e1014274. doi: 10.1371/journal.pcbi.1014274 (PMC13225665; doi:10.1371/journal.pcbi.1014274)

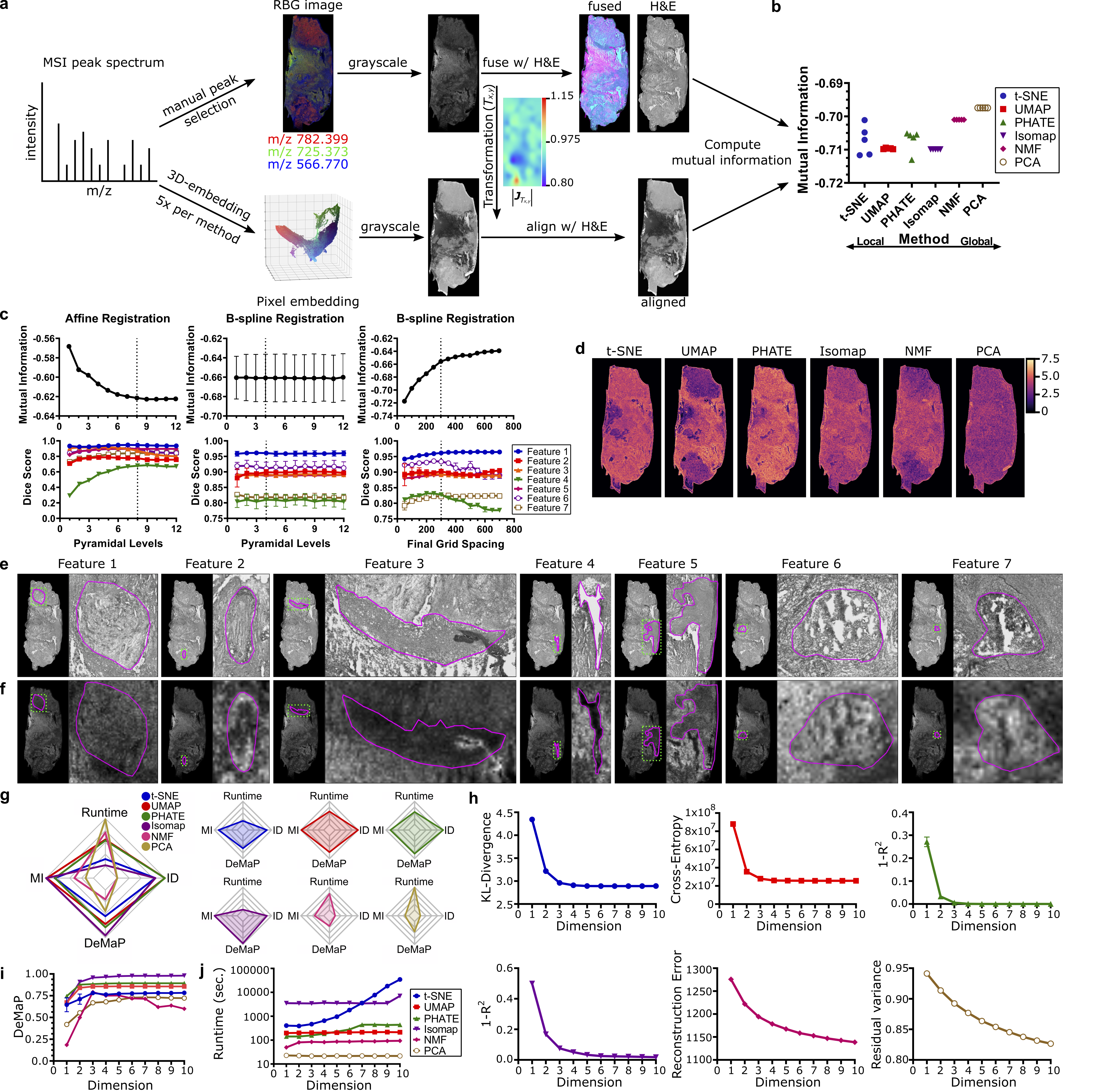

Supplement: S1 Fig — Three mass spectrometry peaks highlighting tissue morphology were manually chosen (top) and were used to create and RGB image representation of the MSI data, which was converted to a grayscale image. The MSI grayscale image was then registered to its corresponding grayscale converted hematoxylin and eosin (H&E) stained section. The deformation field (middle), indicated by the determinant of its spatial Jacobian matrix, was saved to use downstream as a control registration. Three-dimensional Euclidean embeddings of the MSI data were then created using random initializations of each dimension reduction algorithm (bottom). These embeddings were then used to create an RGB image following the procedure above. The spatial transformation created by registering the manually identified peaks with the H&E image was then applied to dimension reduced grayscale images, aligning each to the grayscale H&E image. b. The mutual information between each aligned grayscale embedded image (n = 5 per method) and the grayscale H&E image was calculated using Parzen window histogram density estimation with a histogram bin width of 64. Plot is oriented so that results are consistent with the notion of a “cost function” in optimization contexts, where the goal is to minimize cost. Thus, larger negative values depict higher mutual information. UMAP consistently captures multi-modal information content with respect to the H&E data. c. Optimization of image registration between the grayscale version of manually identified mass spectrometry peaks and the grayscale H&E image (a, top) using mutual information as a cost function with external validation using dice scores on 7 manually annotated regions. Registration parameters used for the final registration used in panel a are indicated with dashed lines. Registration was performed by first aligning images with a multi-resolution affine registration (left). The transformed grayscale version of manually identified mass spectrometry peaks was then re [file pcbi.1014274.s001.tif]

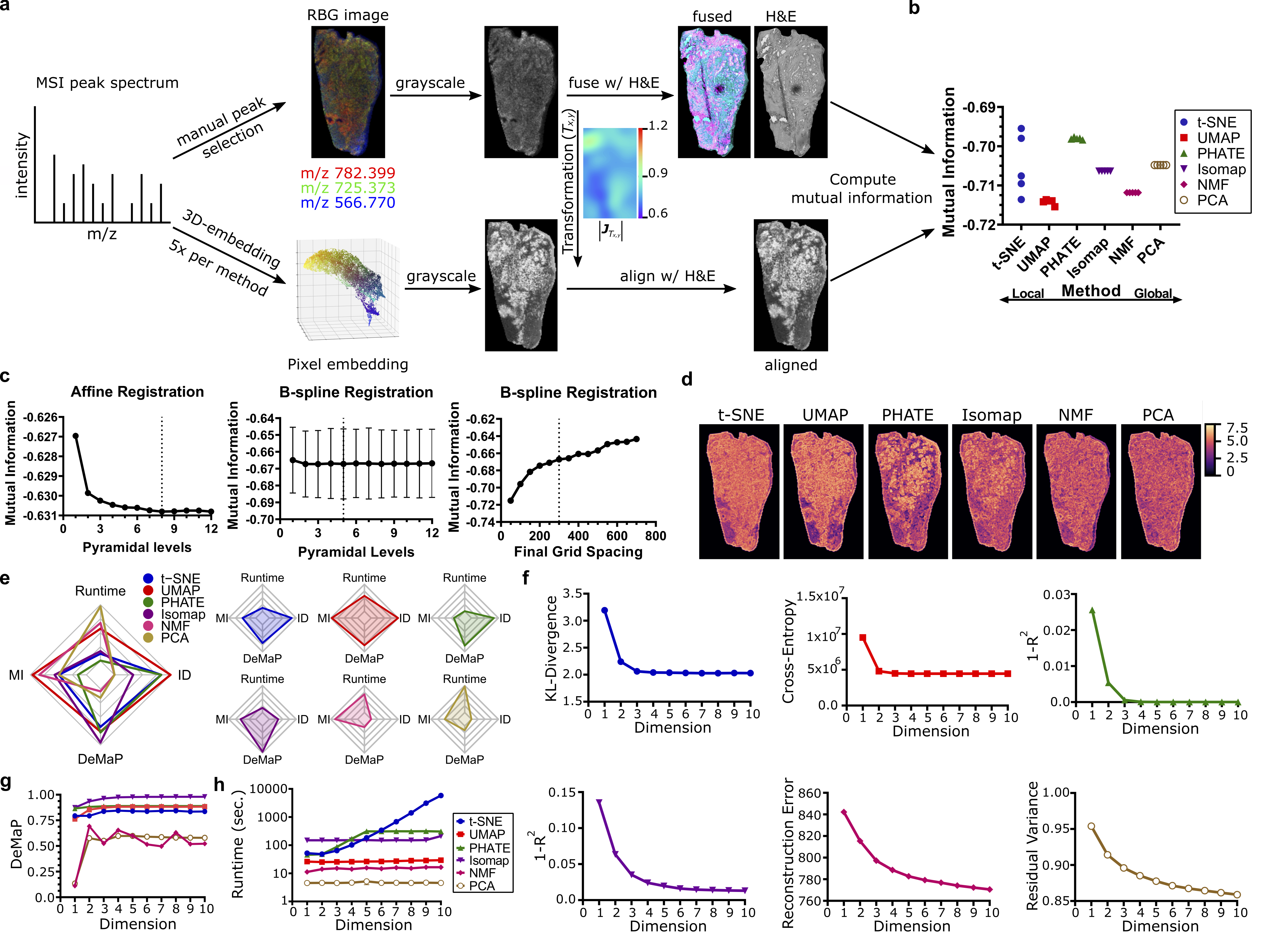

Supplement: S2 Fig — Same as in S1a Fig for prostate cancer tissue biopsy. b. Same as S1b Fig for prostate cancer tissue biopsy. c. Optimization of image registration between the grayscale version of manually identified mass spectrometry peaks and the grayscale H&E image (a, top) using mutual information as a cost function. Registration parameters used for the final registration used in a are indicated with dashed lines. Registration was performed by first aligning images with a multi-resolution affine registration (left). The transformed grayscale version of manually identified mass spectrometry peaks was then registered to the grayscale H&E image using a nonlinear, multi-resolution registration. d. Same as S1d Fig for prostate cancer tissue biopsy. e. Same as S1g Fig for prostate cancer tissue biopsy. f. Same as S1h Fig for prostate cancer tissue biopsy. g. Same as S1i Fig for prostate cancer tissue biopsy. Nonlinear methods Isomap, PHATE, and UMAP all consistently preserve manifold structure without prior filtering of the data with consistent correlations greater than 0.75 across dimensions 2–10. h. Same as S1j Fig for prostate cancer tissue biopsy. (TIF) [file pcbi.1014274.s002.tif]

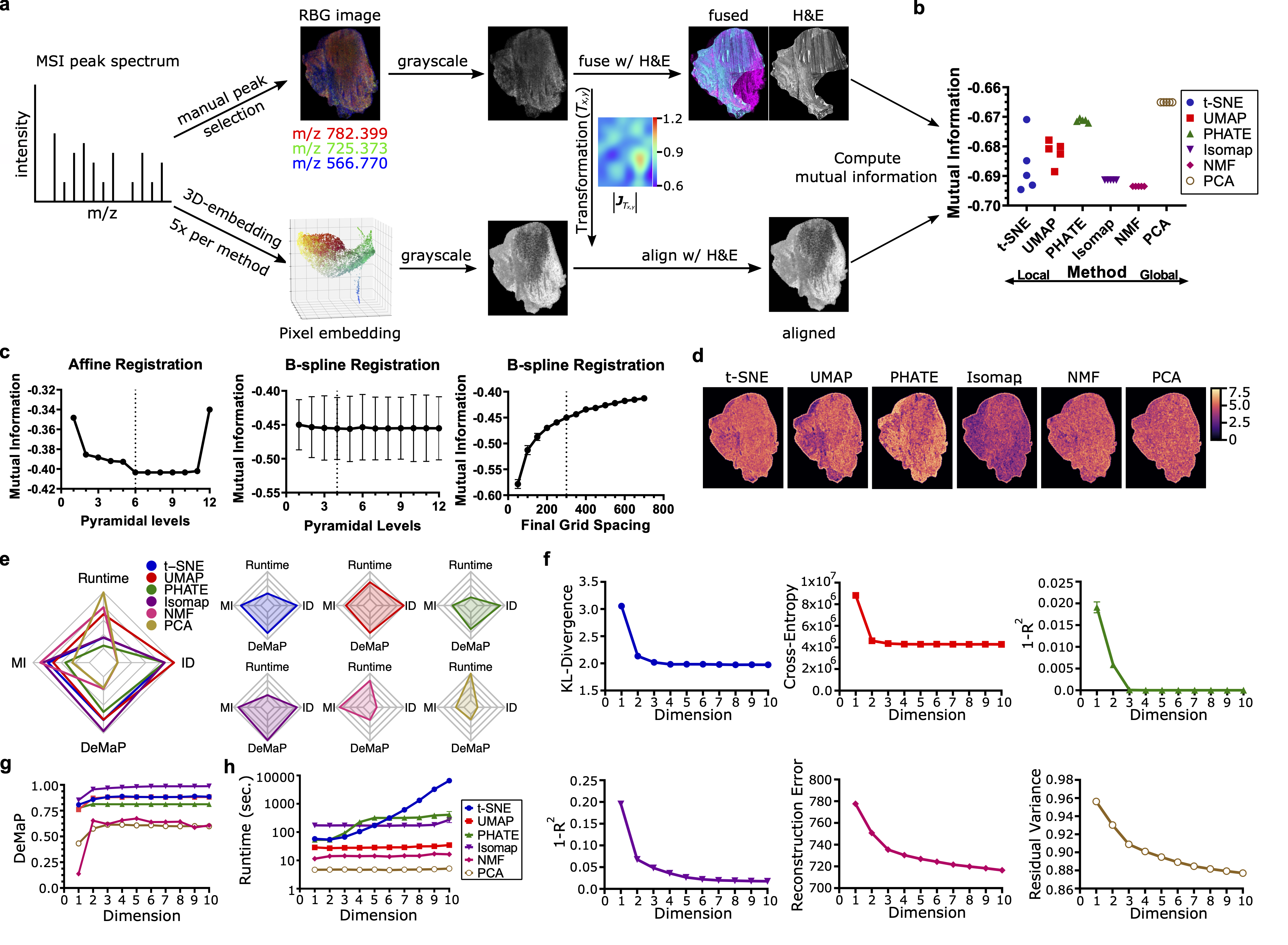

Supplement: S3 Fig — Same as S1 panel a for tonsil tissue biopsy. b. Same as S1 panel b for tonsil tissue biopsy. Isomap and NMF consistently capture multi-modal information content with respect to the H&E data. c. Same as S2c Fig for tonsil tissue biopsy. d. Same as S1d Fig for tonsil tissue biopsy. e. Same as S1g Fig for tonsil tissue biopsy. f. Same as S1h Fig for tonsil tissue biopsy. g. Same as S1l Fig for tonsil tissue biopsy. h. Same as S1j Fig for tonsil tissue biopsy. (TIF) [file pcbi.1014274.s003.tif]

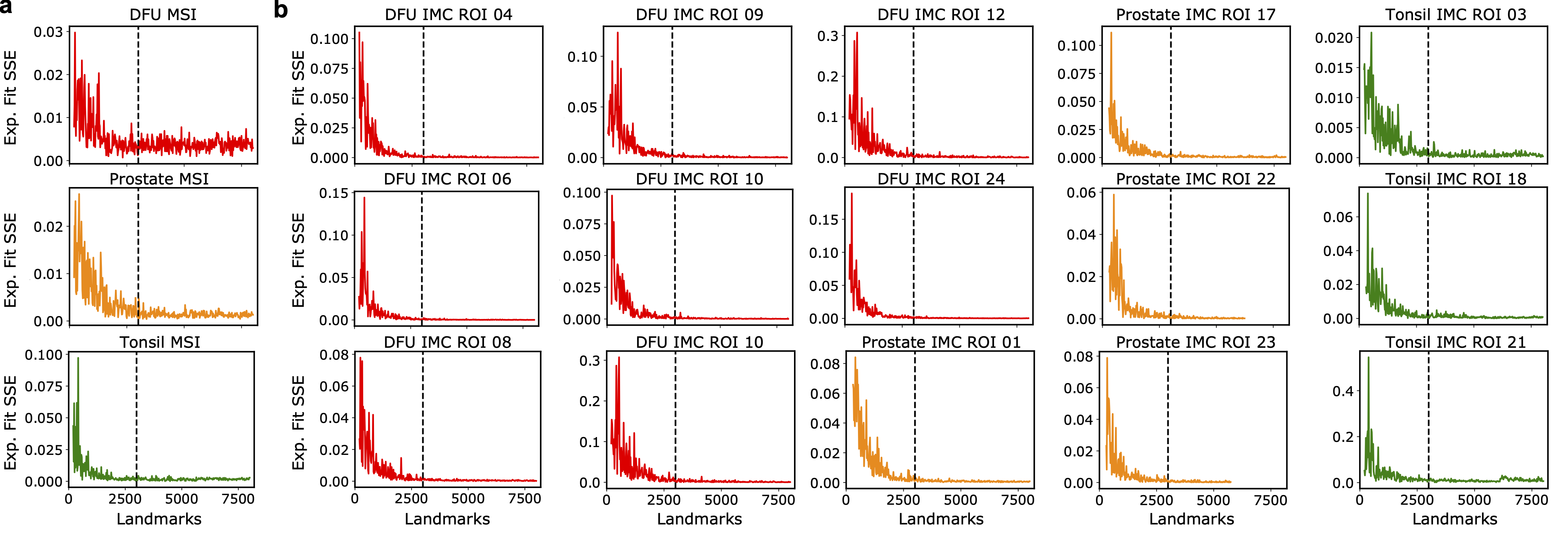

Supplement: S4 Fig — Sum of squared errors of exponential regressions fit to estimated optimal embedding dimensionality selections from spectral landmarks compared to full mass spectrometry imaging data sets across DFU, Prostate, and Tonsil tissues. Discrepancies between exponential regressions fit to the cross-entropy of landmark centroid embeddings and full data set embeddings approach zero as the number of landmarks increases. Dashed lines show MIAAIM’s default selection of 3,000 landmarks for computing manifolds embedding dimensionalities. b. Same as a for subsampled pixels in imaging mass cytometry regions of interest. (TIF) [file pcbi.1014274.s004.tif]

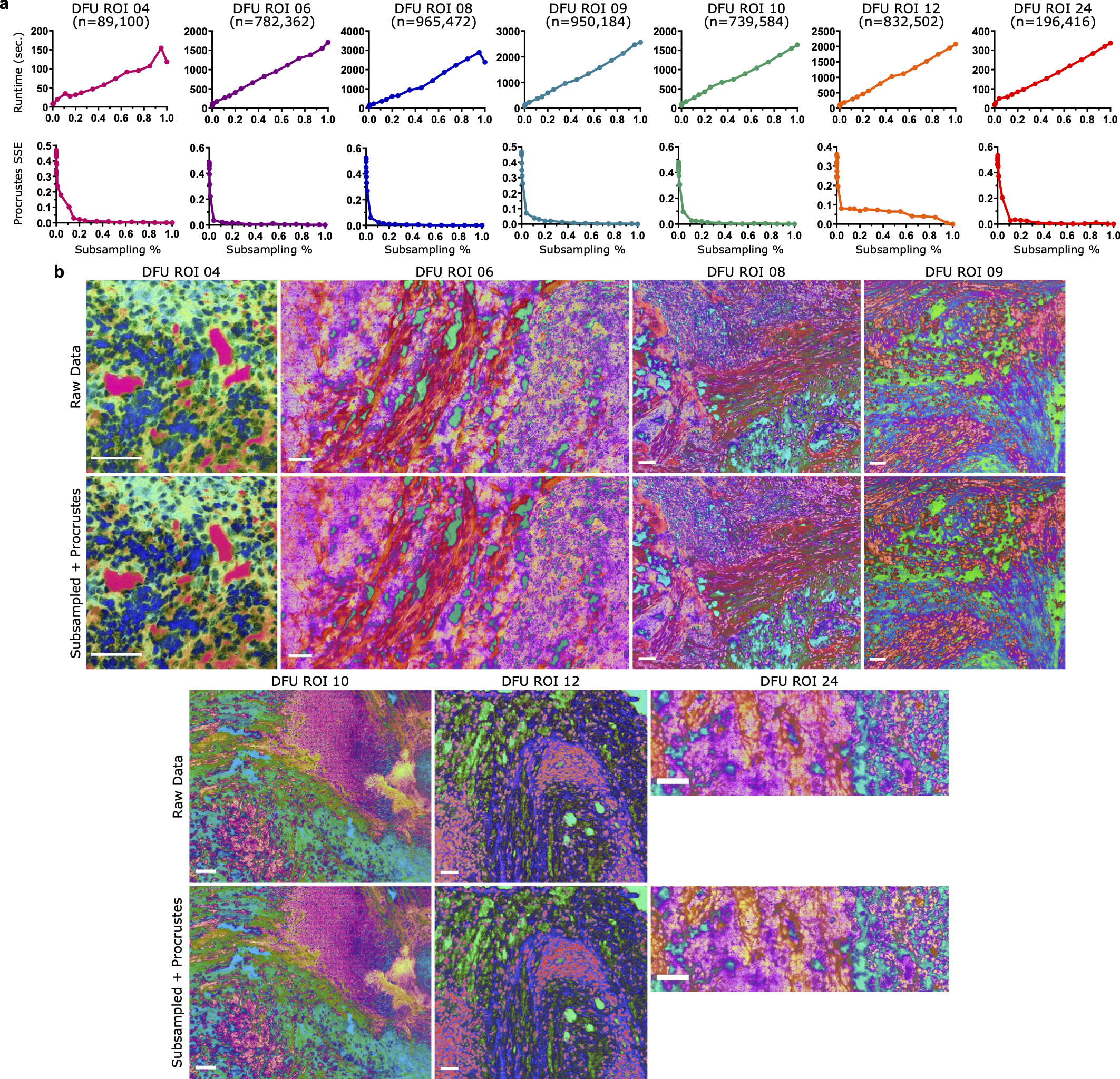

Supplement: S5 Fig — Three-dimensional UMAP embedding (HDIprep compression) runtime plotted with respect to subsampling percentages of IMC ROIs from the DFU tissue biopsy (top). Procrustes transformation sum of squared errors after transforming subsampled embedding to the full pixel embedding across subsampling percentages (bottom). b. Comparison of RGB (red, green, blue) images created by reconstructing images from pixel embeddings on all data (top) versus subsampled data with subsequent out-of-sample projection and Procrustes transformation to align subsampled embedding to the full pixel embedding (bottom) (scale bars = 80 μm). Subsampling percentages of images shown in bottom row of panel b correspond to MIAAIM default parameters that based on number of pixels in images. (TIF) [file pcbi.1014274.s005.tif]

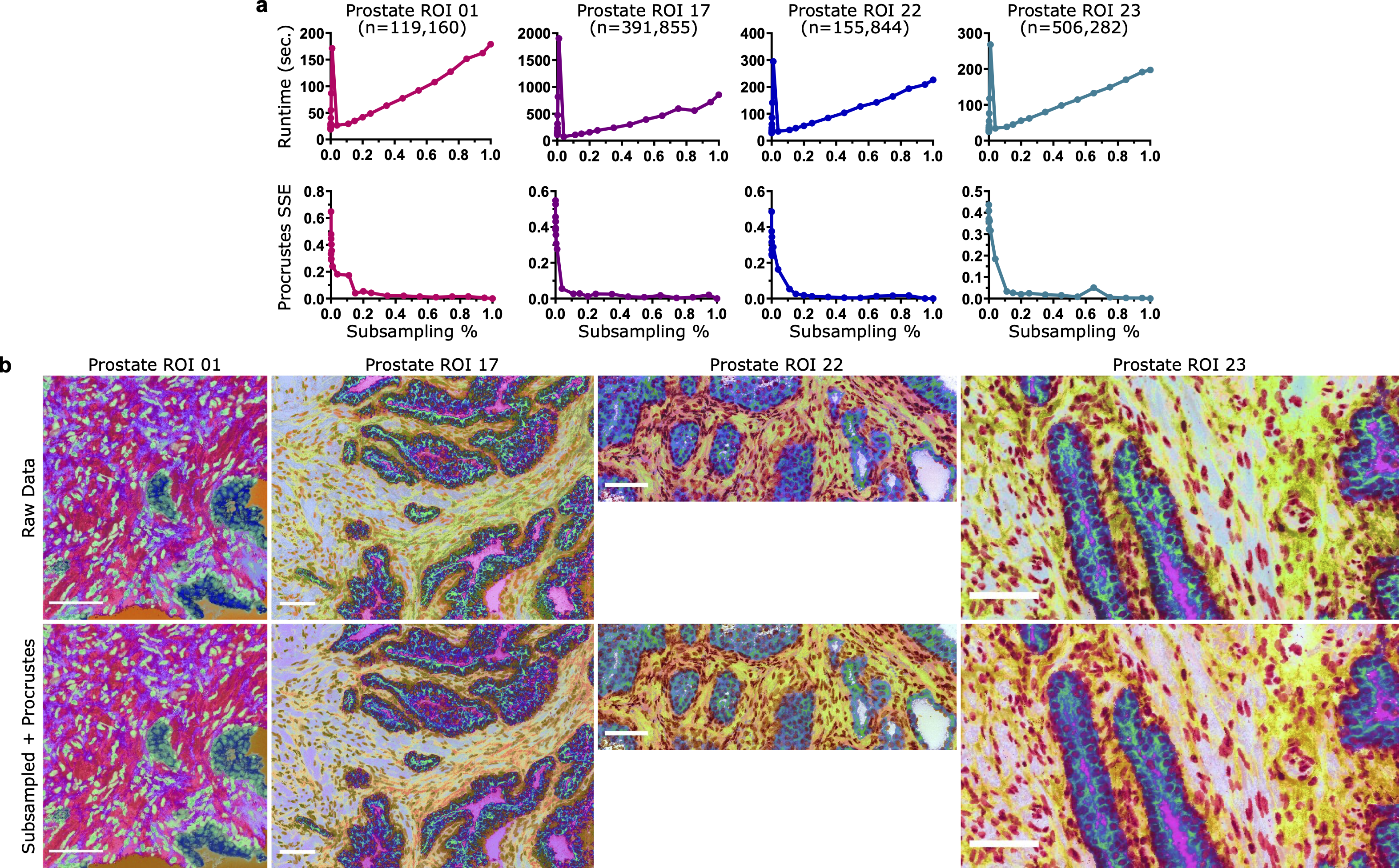

Supplement: S6 Fig — Same as S5 Fig for prostate tumor tissue biopsy IMC ROIs. (TIF) [file pcbi.1014274.s006.tif]

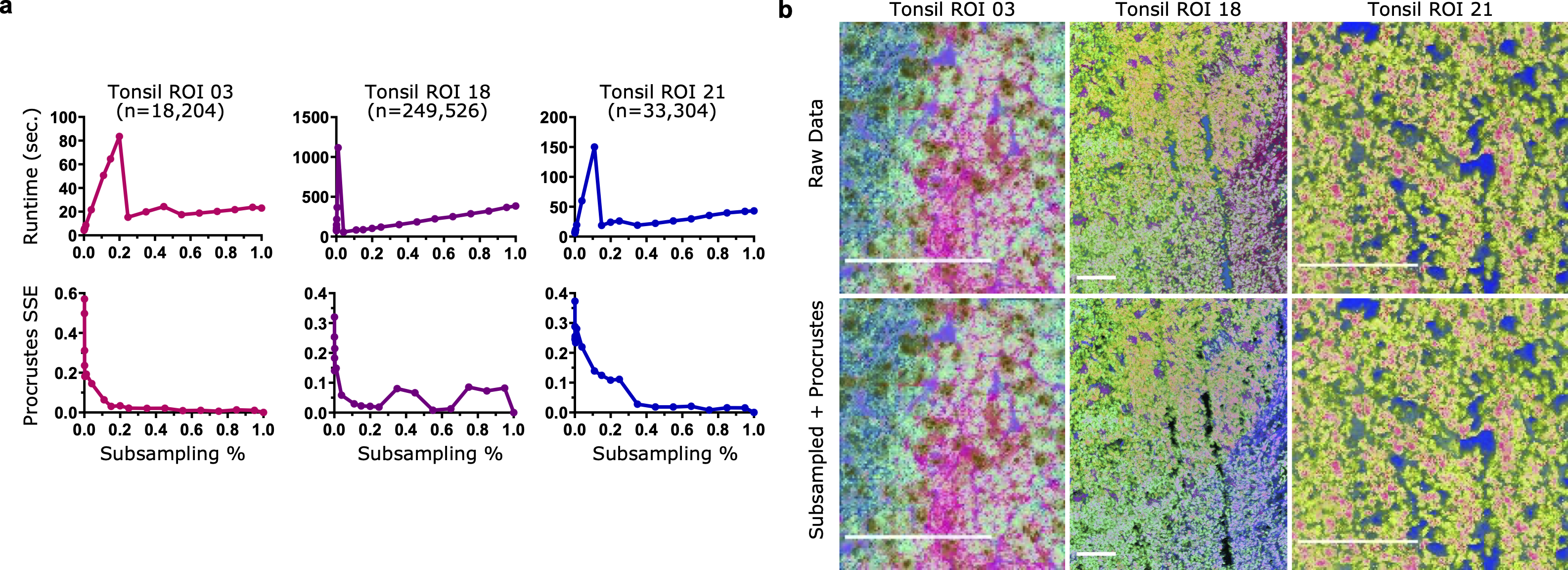

Supplement: S7 Fig — Same as S5 Fig for tonsil tissue biopsy IMC ROIs. (TIF) [file pcbi.1014274.s007.tif]

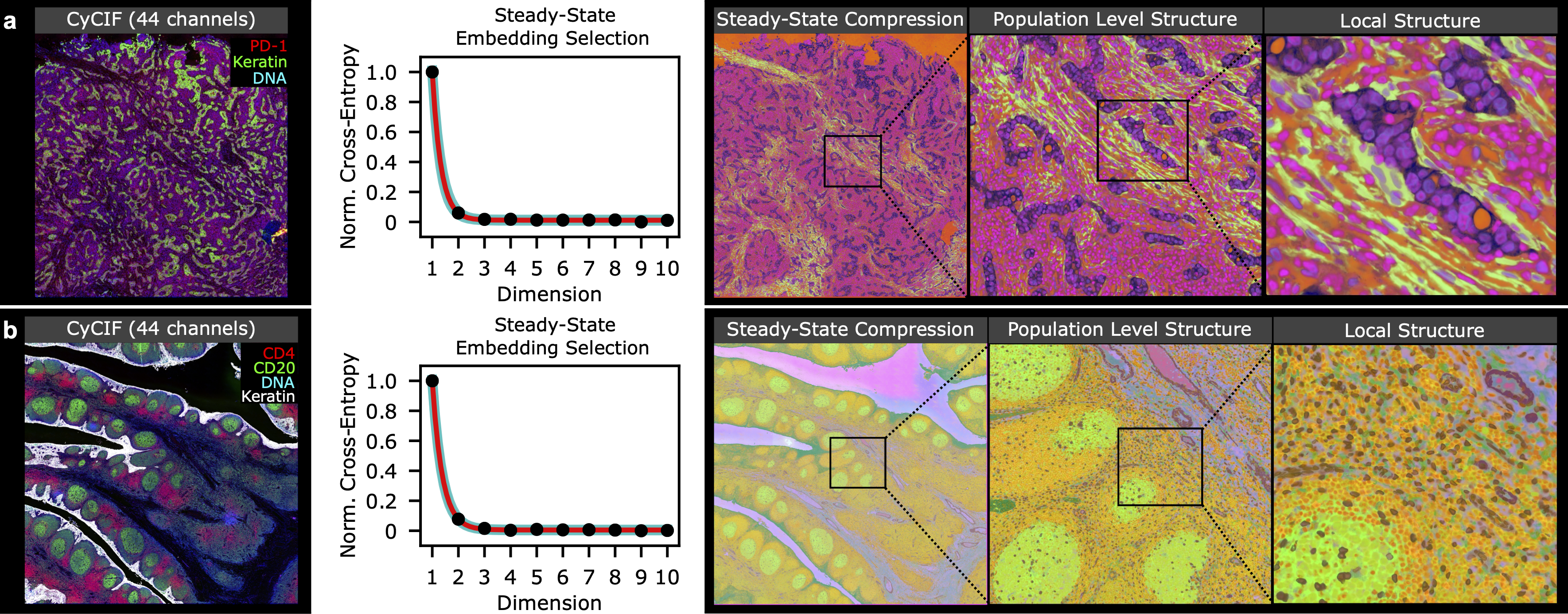

Supplement: S8 Fig — Multiplex CyCIF image of lung adenocarcinoma metastasis to the lymph node (n = ~100 million pixels, 0.65 μm/pixel resolution, 44 channels, 27 antibodies) and corresponding UMAP embedding and spatial reconstruction (shown are three UMAP channels of 4 channel estimated optimal embedding). Parametric UMAP compresses millions of pixels and preserves tissue structure across multiple length scales. b. Same as S8a Fig for tonsil CyCIF data (n = ~256 million pixels, 0.65 μm/pixel resolution). (TIF) [file pcbi.1014274.s008.tif]

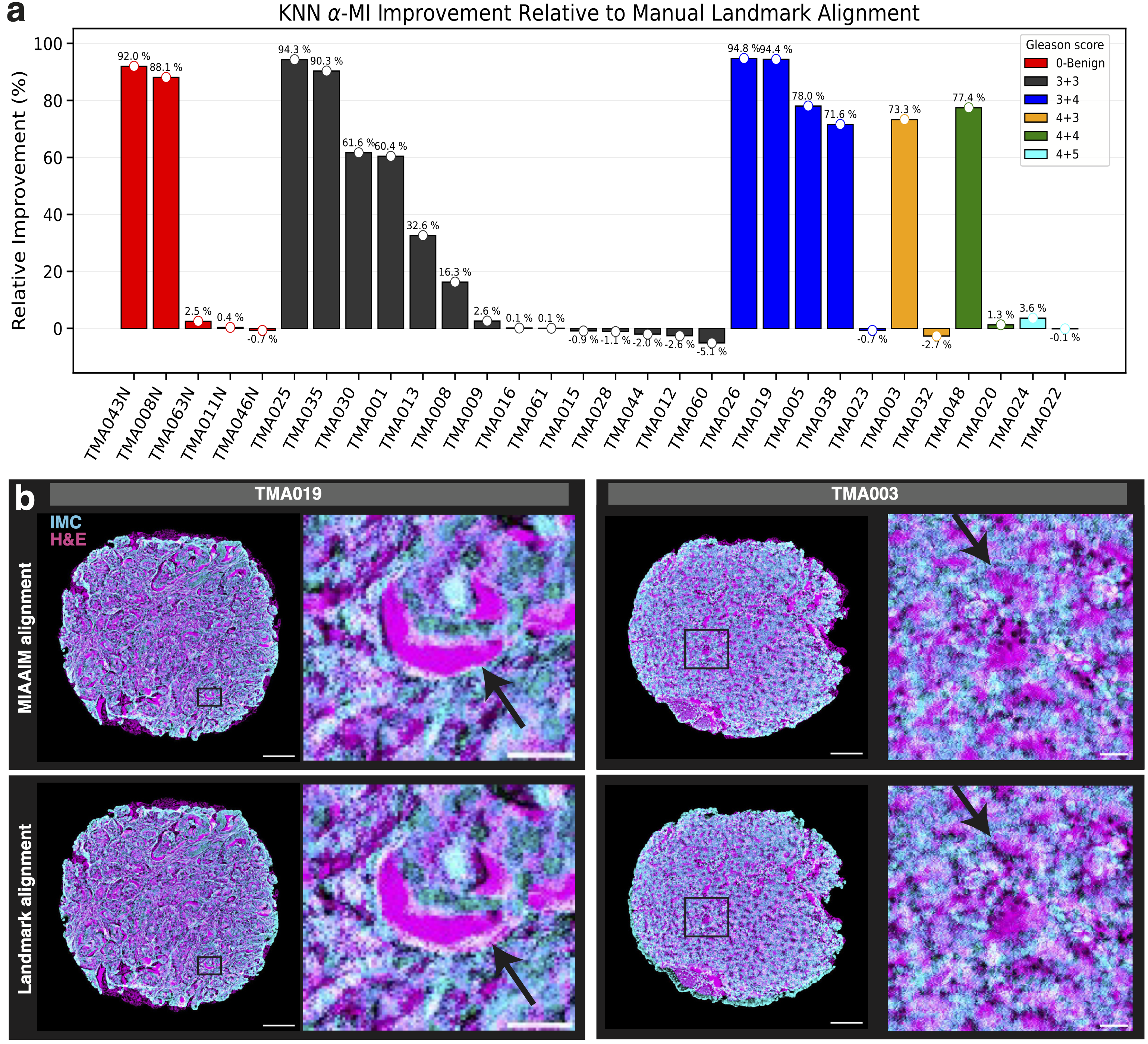

Supplement: S9 Fig — Relative improvement or reduction in α-MI between IMC and MSI modalities for prostate cancer TMA cores registered with MIAAIM versus a manual landmark registration performed using Elastix. b. Visualizations of improvement in alignment using MIAAIM (top) compared to manual landmark alignment (bottom) for two chosen TMA cores (scale bars = 150 μm, 25 μm). Representative UMAP pixel embedding channels and the H&E modality are shown to resolve fine-scale structure. Arrows (insets) point to subtle misalignment resulting from manual landmark registration that is corrected with MIAAIM. (TIF) [file pcbi.1014274.s009.tif]

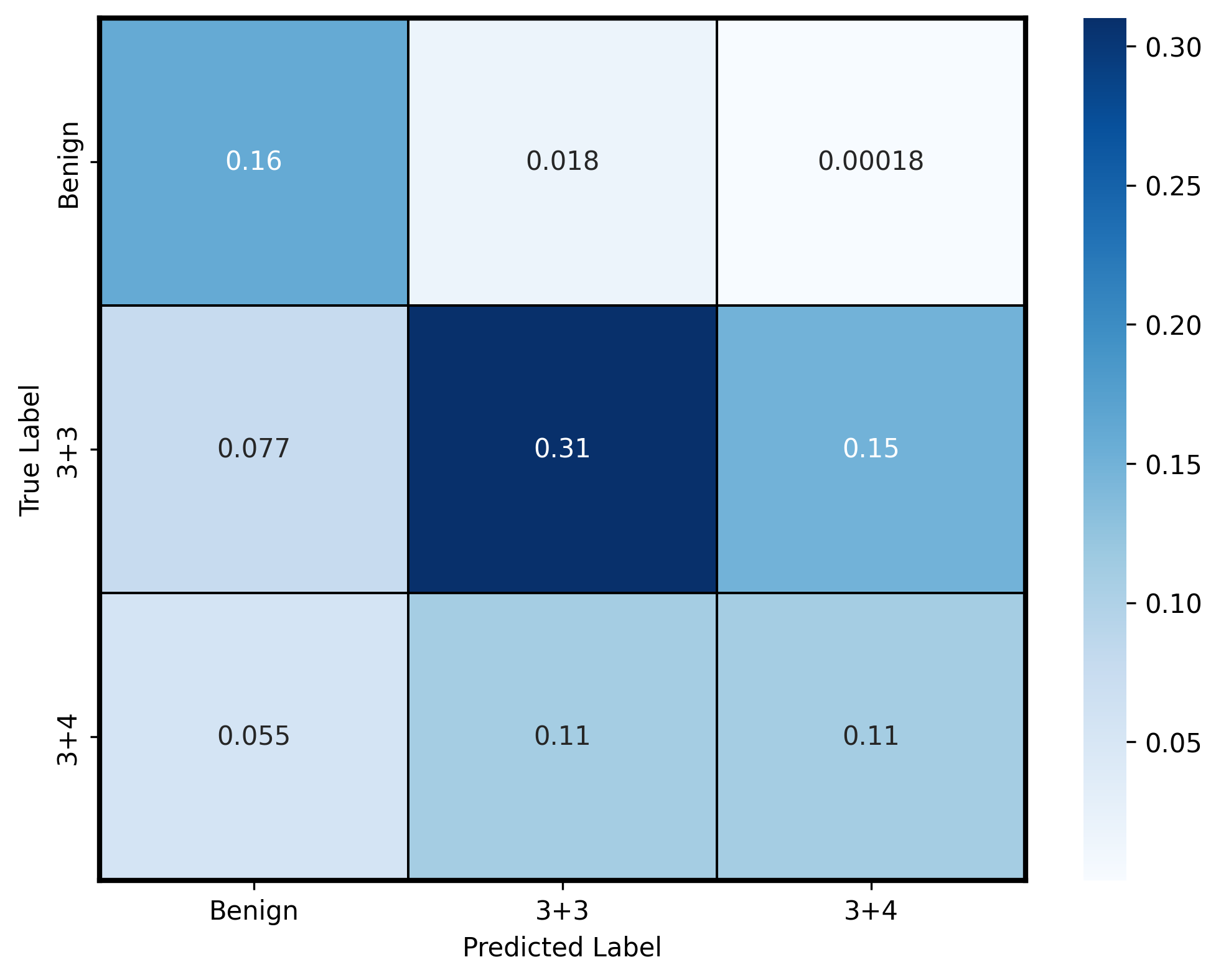

Supplement: S10 Fig — Train and test data were split at the TMA level, and logistic regression was used on single cell and neighborhood interaction measures of IMC/MSI signatures to predict Gleason score. A confusion matrix on classification performance was generated on the test data, showing that classification between benign and cancerous tissue was an easier task compared to classifying between tumor classes. (TIF) [file pcbi.1014274.s010.tif]
