# Supplementary material for: MIAAIM: Multi-omics image integration with dimensional reduction for tissue state mapping
Source: PLoS Comput Biol. 2026 May 26;22(5):e1014274. doi: 10.1371/journal.pcbi.1014274 (PMC13225665; doi:10.1371/journal.pcbi.1014274)
Supplement: S1 Table — (DOCX) [file pcbi.1014274.s011.docx]

**S1 Table | Current imaging technologies anticipated to be compatible and those tested for compatibility with MIAAIM image compression and pre-processing for subsequent alignment**

| **Imaging Technology** | **Input File Format** | **Parameters Detected** | **Tested with MIAAIM** | **Type of Processing** | **Data Set Reference** |
| --- | --- | --- | --- | --- | --- |
| **MSI(1)** | imzML | Proteins, Lipids, Metabolites | ✓ | Compression | This study |
| **Histological Stains (e.g. H&E)** | TIF (F) | Tissue morphology | ✓ | Denoising | This study |
| **IMC(2)** | OME-TIF (F) | Subcellular proteomics | ✓ | Compression | This study |
| **MIBI(3)** | TIF (F) | Subcellular proteomics | ✓ | Compression | (4) |
| **CODEX(5)** | TIF (F) Hyper Stack | Subcellular proteomics | ✓ | Compression | (5) |
| **CyCIF(6)** | OME-TIF (F) | Subcellular proteomics | ✓ | Compression | (7) |
| **4i(8)** | - | Sub-organelle proteomics | 🗶 | Compression | - |
| **Slide-seq(9)** | - | Subcellular Transcriptomics | 🗶 | Compression | - |
| **RNA-ISH related technologies** | - | Transcriptomics | 🗶 | Compression | - |

**Supplementary References**

1. McDonnell LA, Heeren RM. Imaging mass spectrometry. Mass spectrometry reviews. 2007;26(4):606-43.

2. Giesen C, Wang HA, Schapiro D, Zivanovic N, Jacobs A, Hattendorf B, et al. Highly multiplexed imaging of tumor tissues with subcellular resolution by mass cytometry. Nature methods. 2014;11(4):417-22.

3. Angelo M, Bendall SC, Finck R, Hale MB, Hitzman C, Borowsky AD, et al. Multiplexed ion beam imaging of human breast tumors. Nature medicine. 2014;20(4):436.

4. <https://github.com/ionpath/mibilib>.

5. Goltsev Y, Samusik N, Kennedy-Darling J, Bhate S, Hale M, Vazquez G, et al. Deep profiling of mouse splenic architecture with CODEX multiplexed imaging. Cell. 2018;174(4):968-81. e15.

6. Lin J-R, Izar B, Wang S, Yapp C, Mei S, Shah PM, et al. Highly multiplexed immunofluorescence imaging of human tissues and tumors using t-CyCIF and conventional optical microscopes. Elife. 2018;7.

7. Rashid R, Gaglia G, Chen Y-A, Lin J-R, Du Z, Maliga Z, et al. Highly multiplexed immunofluorescence images and single-cell data of immune markers in tonsil and lung cancer. Scientific data. 2019;6(1):1-10.

8. Gut G, Herrmann MD, Pelkmans L. Multiplexed protein maps link subcellular organization to cellular states. Science. 2018;361(6401).

9. Rodriques SG, Stickels RR, Goeva A, Martin CA, Murray E, Vanderburg CR, et al. Slide-seq: A scalable technology for measuring genome-wide expression at high spatial resolution. Science. 2019;363(6434):1463-7.
