# Supplementary material for: MIAAIM: Multi-omics image integration with dimensional reduction for tissue state mapping
Source: PLoS Comput Biol. 2026 May 26;22(5):e1014274. doi: 10.1371/journal.pcbi.1014274 (PMC13225665; doi:10.1371/journal.pcbi.1014274)
Supplement: S3 Table — (DOCX) [file pcbi.1014274.s013.docx]

**S3 Table | Prostate cancer TMA logistic regression model accuracy by modality**

| **Data Modality** | **Accuracy±S.D.** | **Improvement Over Random** |
| --- | --- | --- |
| MSI+MSI interaction + IMC + IMC interaction | 0.6498±0.0738 | 31.65% |
| IMC+IMC interaction | 0.5791±0.0239 | 24.58% |
| IMC Interaction | 0.5694±0.0246 | 23.60% |
| IMC | 0.5574±0.0263 | 22.40% |
| MSI+MSI interaction | 0.5506±0.1448 | 21.72% |
| MSI Interaction | 0.5279±0.1283 | 19.45% |
| MSI | 0.4967±0.0572 | 16.33% |
