# Supplementary material for: MIAAIM: Multi-omics image integration with dimensional reduction for tissue state mapping
Source: PLoS Comput Biol. 2026 May 26;22(5):e1014274. doi: 10.1371/journal.pcbi.1014274 (PMC13225665; doi:10.1371/journal.pcbi.1014274)
Supplement: S2 Note — (DOCX) [file pcbi.1014274.s015.docx]

**S2 Note**

**Notes on the HDIreg workflow’s expected performance**

A fundamental assumption in intensity-based image registration is that quantifiable relationships exist between modalities – this is often met in practice, as shown in our MSI and IMC multi-modal imaging data applications. However, this assumption can be compromised by artefacts, such as folds, tears, and, in the case of serial sectioning, nonlinear deformations. In our experience, glandular tissues, such as those derived from prostate, likely show high structural variability over short distances, making the alignment of images from distinct sections challenging. Manual landmark guidance can be used in difficult use-cases such as those posed by serial tissue sectioning. By using the Elastix library, HDIreg also offers a multitude of similarity measures for single-channel registration, in addition to the manifold alignment scheme used for multichannel registration. We note that histogram-based mutual information has outperformed KNN $\alpha$-MI in these single-channel registration settings (24), which we used in our benchmark studies.

**Supplementary References**

1. McDonnell LA, Heeren RM. Imaging mass spectrometry. Mass spectrometry reviews. 2007;26(4):606-43.

2. Giesen C, Wang HA, Schapiro D, Zivanovic N, Jacobs A, Hattendorf B, et al. Highly multiplexed imaging of tumor tissues with subcellular resolution by mass cytometry. Nature methods. 2014;11(4):417-22.

3. Angelo M, Bendall SC, Finck R, Hale MB, Hitzman C, Borowsky AD, et al. Multiplexed ion beam imaging of human breast tumors. Nature medicine. 2014;20(4):436.

4. <https://github.com/ionpath/mibilib>.

5. Goltsev Y, Samusik N, Kennedy-Darling J, Bhate S, Hale M, Vazquez G, et al. Deep profiling of mouse splenic architecture with CODEX multiplexed imaging. Cell. 2018;174(4):968-81. e15.

6. Lin J-R, Izar B, Wang S, Yapp C, Mei S, Shah PM, et al. Highly multiplexed immunofluorescence imaging of human tissues and tumors using t-CyCIF and conventional optical microscopes. Elife. 2018;7.

7. Rashid R, Gaglia G, Chen Y-A, Lin J-R, Du Z, Maliga Z, et al. Highly multiplexed immunofluorescence images and single-cell data of immune markers in tonsil and lung cancer. Scientific data. 2019;6(1):1-10.

8. Gut G, Herrmann MD, Pelkmans L. Multiplexed protein maps link subcellular organization to cellular states. Science. 2018;361(6401).

9. Rodriques SG, Stickels RR, Goeva A, Martin CA, Murray E, Vanderburg CR, et al. Slide-seq: A scalable technology for measuring genome-wide expression at high spatial resolution. Science. 2019;363(6434):1463-7.

10. Abdelmoula WM, Skraskova K, Balluff B, Carreira RJ, Tolner EA, Lelieveldt BP, et al. Automatic generic registration of mass spectrometry imaging data to histology using nonlinear stochastic embedding. Anal Chem. 2014;86(18):9204-11.

11. Abdelmoula WM, Regan MS, Lopez BGC, Randall EC, Lawler S, Mladek AC, et al. Automatic 3D Nonlinear Registration of Mass Spectrometry Imaging and Magnetic Resonance Imaging Data. Anal Chem. 2019;91(9):6206-16.

12. Li L, Shiradkar R, Gottlieb N, Buzzy C, Hiremath A, Viswanathan VS, et al. Multi-scale statistical deformation based co-registration of prostate MRI and post-surgical whole mount histopathology. Med Phys. 2024;51(4):2549-62.

13. Huizinga W, Poot DH, Guyader JM, Klaassen R, Coolen BF, van Kranenburg M, et al. PCA-based groupwise image registration for quantitative MRI. Med Image Anal. 2016;29:65-78.

14. Guyader JM, Huizinga W, Fortunati V, Poot DHJ, Veenland JF, Paulides MM, et al. Groupwise Multichannel Image Registration. IEEE J Biomed Health Inform. 2019;23(3):1171-80.

15. Klein S, Staring M, Murphy K, Viergever MA, Pluim JP. elastix: a toolbox for intensity-based medical image registration. IEEE Trans Med Imaging. 2010;29(1):196-205.

16. Mahapatra D, Antony B, Sedai S, Garnavi R, editors. Deformable medical image registration using generative adversarial networks. 2018 IEEE 15th International Symposium on Biomedical Imaging (ISBI 2018); 2018 4-7 April 2018.

17. Sorzano CO, Thevenaz P, Unser M. Elastic registration of biological images using vector-spline regularization. IEEE Trans Biomed Eng. 2005;52(4):652-63.

18. Schapiro D, Sokolov A, Yapp C, Muhlich JL, Hess J, Lin J-R, et al. MCMICRO: A scalable, modular image-processing pipeline for multiplexed tissue imaging. bioRxiv. 2021.

19. Schapiro D, Jackson HW, Raghuraman S, Fischer JR, Zanotelli VR, Schulz D, et al. histoCAT: analysis of cell phenotypes and interactions in multiplex image cytometry data. Nature methods. 2017;14(9):873.

20. Berg S, Kutra D, Kroeger T, Straehle CN, Kausler BX, Haubold C, et al. ilastik: Interactive machine learning for (bio) image analysis. Nature Methods. 2019:1-7.

21. Schindelin J, Arganda-Carreras I, Frise E, Kaynig V, Longair M, Pietzsch T, et al. Fiji: an open-source platform for biological-image analysis. Nature methods. 2012;9(7):676-82.

22. Bankhead P, Loughrey MB, Fernández JA, Dombrowski Y, McArt DG, Dunne PD, et al. QuPath: Open source software for digital pathology image analysis. Scientific reports. 2017;7(1):1-7.

23. Sofroniew N, Talley Lambert, Evans, K., Nunez-Iglesias, J., Yamauchi, K., Solak, A. C., Buckley, G., Bokota, G., Tung, T., Ziyangczi, Freeman, J., Boone, P., Winston, P., Loic Royer, Har-Gil, H., Axelrod, S., Rokem, A., Bryant, Hector, Mars Huang, Pranathi Vemuri, Dunham, R., Jakirkham, Siqueira, A. D., Bhavya Chopra, Wood, C., Gohlke, C., Bennett, D., DragaDoncila & Perlman, E. napari/napari: 0.3.5. (Zenodo, 2020). 2020.

24. Staring M, Van Der Heide UA, Klein S, Viergever MA, Pluim JP. Registration of cervical MRI using multifeature mutual information. IEEE transactions on medical imaging. 2009;28(9):1412-21.

25. Maaten Lvd, Hinton G. Visualizing data using t-SNE. Journal of machine learning research. 2008;9(Nov):2579-605.

26. McInnes L, Healy J, Melville J. Umap: Uniform manifold approximation and projection for dimension reduction. arXiv preprint arXiv:180203426. 2018.

27. Moon KR, van Dijk D, Wang Z, Gigante S, Burkhardt DB, Chen WS, et al. Visualizing structure and transitions in high-dimensional biological data. Nature Biotechnology. 2019;37(12):1482-92.

28. Tenenbaum JB, De Silva V, Langford JC. A global geometric framework for nonlinear dimensionality reduction. science. 2000;290(5500):2319-23.

29. Kim H, Park H. Sparse non-negative matrix factorizations via alternating non-negativity-constrained least squares for microarray data analysis. Bioinformatics. 2007;23(12):1495-502.

30. Jolliffe IT, Cadima J. Principal component analysis: a review and recent developments. Philosophical Transactions of the Royal Society A: Mathematical, Physical and Engineering Sciences. 2016;374(2065):20150202.

31. Leland M, John H, Nathaniel S, Lukas G. UMAP: Uniform Manifold Approximation and Projection. Journal of Open Source Software. 2018;3(29):861.

32. Connor Meehan SM, and Wayne Moore. Uniform Manifold Approximation and Projection (UMAP). <https://www.mathworks.com/matlabcentral/fileexchange/71902>, MATLAB Central File Exchange.2020.

33. Ulyanov D. Multicore-tsne. GitHub Repos GitHub. 2016.

34. Pedregosa F, Varoquaux G, Gramfort A, Michel V, Thirion B, Grisel O, et al. Scikit-learn: Machine learning in Python. Journal of machine learning research. 2011;12(Oct):2825-30.

35. Rueckert D, Sonoda LI, Hayes C, Hill DL, Leach MO, Hawkes DJ. Nonrigid registration using free-form deformations: application to breast MR images. IEEE transactions on medical imaging. 1999;18(8):712-21.

36. Lowekamp BC, Chen DT, Ibáñez L, Blezek D. The design of SimpleITK. Frontiers in neuroinformatics. 2013;7:45.

37. Sun K, Marchand-Maillet S, editors. An information geometry of statistical manifold learning. International Conference on Machine Learning; 2014: PMLR.

38. Jayasumana S, Hartley R, Salzmann M, Li H, Harandi M. Kernel methods on Riemannian manifolds with Gaussian RBF kernels. IEEE transactions on pattern analysis and machine intelligence. 2015;37(12):2464-77.

39. Costa JA, Hero AO, editors. Manifold learning using Euclidean k-nearest neighbor graphs [image processing examples]. 2004 IEEE International Conference on Acoustics, Speech, and Signal Processing; 2004: IEEE.

40. Yukich JE. Probability theory of classical Euclidean optimization problems: Springer; 2006.

41. Hero AO, Ma B, Michel OJ, Gorman J. Applications of entropic spanning graphs. IEEE signal processing magazine. 2002;19(5):85-95.

42. Costa JA, Hero AO. Geodesic entropic graphs for dimension and entropy estimation in manifold learning. IEEE Transactions on Signal Processing. 2004;52(8):2210-21.

43. Narayan A, Berger B, Cho H. Assessing single-cell transcriptomic variability through density-preserving data visualization. Nature Biotechnology. 2021:1-10.
