# Supplementary material for: MIAAIM: Multi-omics image integration with dimensional reduction for tissue state mapping
Source: PLoS Comput Biol. 2026 May 26;22(5):e1014274. doi: 10.1371/journal.pcbi.1014274 (PMC13225665; doi:10.1371/journal.pcbi.1014274)
Supplement: S3 Note — (DOCX) [file pcbi.1014274.s016.docx]

**S3 Note**

**HDIprep dimension reduction validation**

**Dimensionality reduction algorithm benchmarking (S1-S3 Figs).**

Our investigation involved a range of dimension reduction methods, spanning from local nonlinear methods, to global, linear methods. Considered methods included t-distributed stochastic neighbor embedding (t-SNE) (25), uniform manifold approximation and projection (UMAP) (26), potential of heat diffusion for affinity-based transition embedding (PHATE) (27), isometric mapping (Isomap) (28), non-negative matrix factorization (NMF) (29), and principal components analysis (PCA) (30).

To assess each methods’ ability to provide an appropriate data representation while enabling multi-modal correspondences, we measured their ability to: (i.) generalize to arbitrary numbers of features or necessary degrees of freedom to accurately represent data modalities, (ii.) succinctly capture data complexity, (iii.) maximize the information content shared between imaging modalities, (iv.) be robust to noise, and (iv.) be computationally efficient.

**(i.-ii.) Estimating intrinsic data dimensionality.** To identify an appropriate method for reducing the complexity of the mass-spectrometry based image data sets, we hypothesized that introducing more degrees of freedom in the coordinates of embedded data (i.e. increases in the dimension of embeddings) would result in increases of the similarity between each methods' embedding and its high-dimensional counterpart with respect to the objective function of each algorithm. Therefore, we viewed each algorithm separately with a distinct objective function and identified the appropriate target dimensionality for the data to be embedded in for each method by analyzing the objective function errors produced by each method after embedding the data in increasing dimensions. To do this, we created a suitable score for estimating the error associated with embedding the MSI data in Euclidean $n$-space, $\mathbb{R}^{n}$, for each dimension reduction method across tissue types and ascending embedding dimensions. For this analysis, we focused on the MSI data rather than the IMC data, which we found was not feasible to apply most dimension reduction methods to because of data size (number of pixels/high resolution).

To determine each method's estimated intrinsic dimensionality of the data set, we identified the point in each methods’ error graph where increases in dimensionality no longer reduced embedding error. To do this, we viewed increases in the dimensionality of real-valued data in a natural way by modelling increases in dimensionality as exponential increases in potential positions of points (i.e., increasing copies of the real line, $\mathbb{R}^{n}$). We therefore fit a least-squares exponential regression to the error curves of data embedding, and 95% confidence intervals (CI) were constructed by modelling gaussian residual processes. The optimal embedding dimensions for each method were selected by simulating samples along the expected value of the fit curve and identifying the first integer-valued instance that fell within the 95% CI for the exponential asymptote. In this way, the minimum degrees of freedom necessary to capture data complexity was identified. The average error curves for each method across 5 random initializations of each algorithm across each MSI data set are shown in **S1, S2, S3 Figs.** The methods and rationale used for calculating each method's embedding error are outlined below:

UMAP. The UMAP algorithm falls in the category of manifold learning techniques, and it aims to optimize the embedding of a fuzzy simplicial set representation of high-dimensional data into lower dimensional Euclidean spaces. Practically, a low dimensional fuzzy simplicial set is optimized so that the fuzzy set cross-entropy between its high-dimensional counterpart is minimized. The fuzzy-set cross entropy is defined explicitly in **Definition 1, Methods**, given by McInnes and Healy (26).

While the theoretical underpinnings of UMAP are grounded in category theory, the practical implementation of UMAP boils down to weighted graphs. To provide an estimate of the intrinsic dimensionality of the data determined by UMAP, we used the open-source implementation in Python (31) with 15 nearest neighbors, a value of 0.1 for minimum distance in the resulting embedding, and we allow the algorithm to optimize the embedding for the default value of 200 iterations for each dimension. The cross-entropy for each dimension between the high dimensional fuzzy simplicial set and the low dimensional counterpart was computed using a Python-converted module of the MATLAB UMAP implementation (32).

T-SNE. T-SNE is a manifold-based dimension reduction method that aims to preserve local structure in data sets for visualization purposes (25). To achieve this, t-SNE minimizes the difference between distributions representing the local similarity between points in the original, high-dimensional ambient space and the respective low dimensional embedding. The difference between these two distributions is determined by the Kullback-Leibler (KL) divergence between them. As a result, we report the final value of the KL-divergence upon embedding as a means of estimating the error associated with t-SNE embeddings in each dimension. For all t-SNE calculations, we use an open-source multi-core implementation (33) with the default parameters (perplexity of 30).

Isomap. Isomap is a manifold-based dimension reduction method that uses classic multidimensional scaling (MDS) to preserve interpoint geodesic distances (28). To do this, the geodesic distance between points are determined by shortest-path graph distances using the Euclidean metric. The pairwise distance matrix represented by this graph is then embedded into $n$-dimensional Euclidean space via classical MDS, a metric-preserving technique that finds the optimal transformation for inter-point Euclidean metric preservation. As a result of the implicit linearity in classic MDS, we estimate the intrinsic dimensionality of the data by calculating the reconstruction error in each dimension using $1-R^{2}$, where $R$ is the standard linear correlation coefficient between the geodesic distance matrix and the pairwise Euclidean distance matrix in $\mathbb{R}^{n}$. For all calculations, 15 nearest neighbors were chosen for determining shortest-path graph distances, and the Minkowski metric with an order of two for the norm of the difference ${|\left| u-v \right||}_{p}$ was chosen. All Isomap calculations were performed using Scikit-learn (34).

PHATE*.* PHATE is a manifold-based dimension reduction technique developed for data visualization that captures both global and local features of data sets (27). PHATE achieves this by modelling relationships between data points as t-step random walk diffusion probabilities and by subsequently calculating potential distances between data points through comparison of each pair of points' respective diffusion distributions to all others in the data set (27). These potential distances are then embedded in $n$-dimensional space using classic MDS followed by metric MDS. Metric MDS is suitable for embedding points with dissimilarities given by any metric, relaxing Euclidean constraints imposed by classical MDS, through minimizing the following stress function $S$:

$$S\left( \hat{x}_{1}\ldots\hat{x}_{N} \right)=\sqrt{\frac{\sum_{i,j} \left( D_{x_{i},x_{j}}-|\left| \hat{x}_{i}-\hat{x}_{j} \right|| \right)^{2}}{\sum_{i,j} \left( D_{x_{i},x_{j}} \right)^{2}}}$$

where $D$ is the metric defined over points $x_{1}\ldots x_{N}$ in the original data set, and $\hat{x}_{1}\ldots\hat{x}_{N}\epsilon\mathbb{R}^{n}$ are the corresponding embedded data points in dimension $n$. This stress function amounts to a least-squares optimization problem. In the scalable form of PHATE used for large data sets, landmarks instead of points are embedded in $n$-dimensional Euclidean space based on their pairwise potential distances using the above stress function. Out-of-sample embedding for all data points is performed by calculating linear combinations of the t-step transition matrix from points to landmarks using the embedded landmark coordinates as weights. If the stress function for metric MDS is zero, then the dimension reduction process is fully able to embed and capture the interpoint distances of the data. This would provide an error estimate to be used for analyses on intrinsic data dimension for the full data set and full PHATE algorithm; however, for the landmark-based calculations, not all points are embedded using metric MDS. Given the linear interpolation scheme and the initialization of scalable PHATE using classical MDS on aa potential distances, we posited that the reconstruction error given by $1-R^{2}$, where $R$ is the linear correlation coefficient between the point-to-landmark transition matrix and the pairwise Euclidean distance matrix in $\mathbb{R}^{n}$, provides an estimate for the error associated with embedding the full data set. All PHATE calculations were performed in Python using 15 nearest neighbors and the default number of 2,000 landmark points.

NMF*.* Non-negative matrix factorization (NMF) (29) is a linear dimension reduction technique that aims to minimize the divergence between an input matrix $\boldsymbol{X}$and its reconstruction obtained through the matrix factorization $\boldsymbol{WH}$. Through this factorization, linear combinations of the columns of $\boldsymbol{W}$ are produced using weights from $\boldsymbol{H.}$The Frobenius norm between $\boldsymbol{X}$ and $\boldsymbol{WH}$ was used in our calculations, with the divergence between the two being calculated as $\frac{1}{2}\left| \left| \boldsymbol{X-WH} \right| \right|_{2}$. Thus, to estimate the error associated with each embedding dimension, this divergence or reconstruction error was plotted. For all calculations, each channel in the data set was min-max rescaled to a 0 to 1 range to ensure that only positive elements were included in $\boldsymbol{X}$. All calculations were performed using Scikit-learn (34).

PCA. Principal components analysis (PCA) is a linear dimension reduction method that aims to capture the primary axes of variation in the data on a global level (30). To determine the intrinsic dimensionality of the data set estimated by PCA, the cumulative percentage of residual variance remaining after dimension reduction for each component is plotted. Given a component $1\leq d\leq n-1$ where $n$ is the number of dimensions of the original data set, the percentage of variance explained by embedding in dimension $d$ is determined by summing the $d$-largest eigenvalues of the covariance matrix of the full data set. For all calculations, each channel in the data set was standardized by removing the mean and scaling to unit variance. Standardization was used to ensure that no feature dominated the objective function of PCA. All calculations were performed using Scikit-learn (34).

**(iii.) Assessing information content relative to H&E tissue morphology***.* In order to have an unbiased assessment of image to image information content between embedded data produced from each dimension reduction method and corresponding H&E stained tissue biopsy sections, three channels from MSI data were carefully chosen as representative peaks that highlighted morphological characteristics of the tissue (*m/z* peaks 782.399, 725.373, 566.770 for diabetic foot ulcer, prostate, and tonsil), a hyperspectral image was created, converted to gray scale, and was registered to the corresponding gray scale converted H&E image (**S1a, S2a, S3a Figs**).

To ensure an appropriate alignment between the manually chosen gray scale MSI image of the diabetic foot ulcer and the gray scale H&E image, the mutual information of the registration and the dice score of seven paired ROIs between the two images were assessed across hyper-parameter grids for an initial affine registration and subsequent nonlinear registration (**S1c Fig**). For the prostate and tonsil tissues, we optimized the mutual information alone (**S2c, 23c Figs**). The results across hyper-parameter grids were then analyzed to choose the optimal parameters for each step in the registration scheme.

For the affine registrations, the hyper-parameter search resulted in a chosen number of resolutions in the multi-resolution pyramidal hierarchy. For the nonlinear registrations, both the number of resolutions and final uniform grid-spacing for the B-spline controls points were determined by the hyper-parameter grid search. In both registrations, the number of resolutions either improved registration results or left the registration unchanged. However, during the nonlinear registration, finer control point grid-spacing schedules resulted in improved registrations indicated by the mutual information, yet they resulted in regions with unrealistic warping even with the addition of regularization using deformation bending energy penalties (35). A value of 300 for the final grid-spacing was chosen as a balance between improved registration indicated by the cost function and increased warping.

The resulting deformation field was then applied to the gray scale hyperspectral images created from each dimension reduction algorithm to spatially align them equally with the H&E images of each tissue. Prior to calculating the mutual information between the H&E and embedded MSI images, a nonzero intersection was applied to the pair of images. The nonzero intersection was used to account for any edge effects introduced in the registration by using three manually chosen MSI peaks, which could have adversely affected the registration and mutual information calculations in our analysis if they were not well-represented at all locations in the images. The mutual information between each registered dimension reduction image (n = 5 per method) was then calculated using a Parzen window-based method in SimpleITK (36) (**S1b, S2b, S3b Figs.**).

**(iv.) Assessing algorithm robustness to noise.** Through the assessment of data intrinsic dimensionality, we learned that both high-dimensional imaging modalities (MSI and IMC) follow a manifold structure, where the dimensionality of the data can be approximated with fewer degrees of freedom than the number of parameters initially given in the ambient space. Using this information, in addition to the visual quality of subsequent spatial mappings of each method back onto tissues, as evidence to justify the assumption of such manifold structure, we then proceeded to interrogate the ability of each algorithm to preserve geodesic distances in low dimensional embeddings with and without the addition of "noisy" peaks and/or technical variation.

To do this, we utilized the denoised manifold preservation (DEMaP) metric (27). By computing the DEMaP metric (Spearman's rank correlation coefficient) between geodesic distances in the ambient space of a peak-picked MSI data set and the pairwise embedded Euclidean distances between data points from the corresponding non-peak-picked data set, we assessed the ability of each algorithm to preserve the manifold structure of the data set in the presence of noise. Since all the algorithms used were either calculated using the Euclidean metric with 15 nearest neighbors or they inherently assume a Euclidean structure, we calculated geodesic distances in the peak picked MSI data set using 15 nearest neighbors using the Euclidean metric. Peak-picking was performed in SCiLS Lab 2018b using orthogonal matching pursuit with a maximum number of peaks of 1,000. The DEMaP scores for each method across 5 random initializations of each algorithm for each MSI data set are shown in **S1g, S2g, S3g Figs**.

**(v.) Assessing computational runtime.** Computational runtime for all methods was captured across 5 randomly initialized runs for each algorithm for embedding dimensions 1-10 across diabetic foot ulcer, prostate cancer, and tonsil tissue biopsy MSI data (**S1h, S2h, S3h Figs**).

**Supplementary References**

1. McDonnell LA, Heeren RM. Imaging mass spectrometry. Mass spectrometry reviews. 2007;26(4):606-43.

2. Giesen C, Wang HA, Schapiro D, Zivanovic N, Jacobs A, Hattendorf B, et al. Highly multiplexed imaging of tumor tissues with subcellular resolution by mass cytometry. Nature methods. 2014;11(4):417-22.

3. Angelo M, Bendall SC, Finck R, Hale MB, Hitzman C, Borowsky AD, et al. Multiplexed ion beam imaging of human breast tumors. Nature medicine. 2014;20(4):436.

4. <https://github.com/ionpath/mibilib>.

5. Goltsev Y, Samusik N, Kennedy-Darling J, Bhate S, Hale M, Vazquez G, et al. Deep profiling of mouse splenic architecture with CODEX multiplexed imaging. Cell. 2018;174(4):968-81. e15.

6. Lin J-R, Izar B, Wang S, Yapp C, Mei S, Shah PM, et al. Highly multiplexed immunofluorescence imaging of human tissues and tumors using t-CyCIF and conventional optical microscopes. Elife. 2018;7.

7. Rashid R, Gaglia G, Chen Y-A, Lin J-R, Du Z, Maliga Z, et al. Highly multiplexed immunofluorescence images and single-cell data of immune markers in tonsil and lung cancer. Scientific data. 2019;6(1):1-10.

8. Gut G, Herrmann MD, Pelkmans L. Multiplexed protein maps link subcellular organization to cellular states. Science. 2018;361(6401).

9. Rodriques SG, Stickels RR, Goeva A, Martin CA, Murray E, Vanderburg CR, et al. Slide-seq: A scalable technology for measuring genome-wide expression at high spatial resolution. Science. 2019;363(6434):1463-7.

10. Abdelmoula WM, Skraskova K, Balluff B, Carreira RJ, Tolner EA, Lelieveldt BP, et al. Automatic generic registration of mass spectrometry imaging data to histology using nonlinear stochastic embedding. Anal Chem. 2014;86(18):9204-11.

11. Abdelmoula WM, Regan MS, Lopez BGC, Randall EC, Lawler S, Mladek AC, et al. Automatic 3D Nonlinear Registration of Mass Spectrometry Imaging and Magnetic Resonance Imaging Data. Anal Chem. 2019;91(9):6206-16.

12. Li L, Shiradkar R, Gottlieb N, Buzzy C, Hiremath A, Viswanathan VS, et al. Multi-scale statistical deformation based co-registration of prostate MRI and post-surgical whole mount histopathology. Med Phys. 2024;51(4):2549-62.

13. Huizinga W, Poot DH, Guyader JM, Klaassen R, Coolen BF, van Kranenburg M, et al. PCA-based groupwise image registration for quantitative MRI. Med Image Anal. 2016;29:65-78.

14. Guyader JM, Huizinga W, Fortunati V, Poot DHJ, Veenland JF, Paulides MM, et al. Groupwise Multichannel Image Registration. IEEE J Biomed Health Inform. 2019;23(3):1171-80.

15. Klein S, Staring M, Murphy K, Viergever MA, Pluim JP. elastix: a toolbox for intensity-based medical image registration. IEEE Trans Med Imaging. 2010;29(1):196-205.

16. Mahapatra D, Antony B, Sedai S, Garnavi R, editors. Deformable medical image registration using generative adversarial networks. 2018 IEEE 15th International Symposium on Biomedical Imaging (ISBI 2018); 2018 4-7 April 2018.

17. Sorzano CO, Thevenaz P, Unser M. Elastic registration of biological images using vector-spline regularization. IEEE Trans Biomed Eng. 2005;52(4):652-63.

18. Schapiro D, Sokolov A, Yapp C, Muhlich JL, Hess J, Lin J-R, et al. MCMICRO: A scalable, modular image-processing pipeline for multiplexed tissue imaging. bioRxiv. 2021.

19. Schapiro D, Jackson HW, Raghuraman S, Fischer JR, Zanotelli VR, Schulz D, et al. histoCAT: analysis of cell phenotypes and interactions in multiplex image cytometry data. Nature methods. 2017;14(9):873.

20. Berg S, Kutra D, Kroeger T, Straehle CN, Kausler BX, Haubold C, et al. ilastik: Interactive machine learning for (bio) image analysis. Nature Methods. 2019:1-7.

21. Schindelin J, Arganda-Carreras I, Frise E, Kaynig V, Longair M, Pietzsch T, et al. Fiji: an open-source platform for biological-image analysis. Nature methods. 2012;9(7):676-82.

22. Bankhead P, Loughrey MB, Fernández JA, Dombrowski Y, McArt DG, Dunne PD, et al. QuPath: Open source software for digital pathology image analysis. Scientific reports. 2017;7(1):1-7.

23. Sofroniew N, Talley Lambert, Evans, K., Nunez-Iglesias, J., Yamauchi, K., Solak, A. C., Buckley, G., Bokota, G., Tung, T., Ziyangczi, Freeman, J., Boone, P., Winston, P., Loic Royer, Har-Gil, H., Axelrod, S., Rokem, A., Bryant, Hector, Mars Huang, Pranathi Vemuri, Dunham, R., Jakirkham, Siqueira, A. D., Bhavya Chopra, Wood, C., Gohlke, C., Bennett, D., DragaDoncila & Perlman, E. napari/napari: 0.3.5. (Zenodo, 2020). 2020.

24. Staring M, Van Der Heide UA, Klein S, Viergever MA, Pluim JP. Registration of cervical MRI using multifeature mutual information. IEEE transactions on medical imaging. 2009;28(9):1412-21.

25. Maaten Lvd, Hinton G. Visualizing data using t-SNE. Journal of machine learning research. 2008;9(Nov):2579-605.

26. McInnes L, Healy J, Melville J. Umap: Uniform manifold approximation and projection for dimension reduction. arXiv preprint arXiv:180203426. 2018.

27. Moon KR, van Dijk D, Wang Z, Gigante S, Burkhardt DB, Chen WS, et al. Visualizing structure and transitions in high-dimensional biological data. Nature Biotechnology. 2019;37(12):1482-92.

28. Tenenbaum JB, De Silva V, Langford JC. A global geometric framework for nonlinear dimensionality reduction. science. 2000;290(5500):2319-23.

29. Kim H, Park H. Sparse non-negative matrix factorizations via alternating non-negativity-constrained least squares for microarray data analysis. Bioinformatics. 2007;23(12):1495-502.

30. Jolliffe IT, Cadima J. Principal component analysis: a review and recent developments. Philosophical Transactions of the Royal Society A: Mathematical, Physical and Engineering Sciences. 2016;374(2065):20150202.

31. Leland M, John H, Nathaniel S, Lukas G. UMAP: Uniform Manifold Approximation and Projection. Journal of Open Source Software. 2018;3(29):861.

32. Connor Meehan SM, and Wayne Moore. Uniform Manifold Approximation and Projection (UMAP). <https://www.mathworks.com/matlabcentral/fileexchange/71902>, MATLAB Central File Exchange.2020.

33. Ulyanov D. Multicore-tsne. GitHub Repos GitHub. 2016.

34. Pedregosa F, Varoquaux G, Gramfort A, Michel V, Thirion B, Grisel O, et al. Scikit-learn: Machine learning in Python. Journal of machine learning research. 2011;12(Oct):2825-30.

35. Rueckert D, Sonoda LI, Hayes C, Hill DL, Leach MO, Hawkes DJ. Nonrigid registration using free-form deformations: application to breast MR images. IEEE transactions on medical imaging. 1999;18(8):712-21.

36. Lowekamp BC, Chen DT, Ibáñez L, Blezek D. The design of SimpleITK. Frontiers in neuroinformatics. 2013;7:45.

37. Sun K, Marchand-Maillet S, editors. An information geometry of statistical manifold learning. International Conference on Machine Learning; 2014: PMLR.

38. Jayasumana S, Hartley R, Salzmann M, Li H, Harandi M. Kernel methods on Riemannian manifolds with Gaussian RBF kernels. IEEE transactions on pattern analysis and machine intelligence. 2015;37(12):2464-77.

39. Costa JA, Hero AO, editors. Manifold learning using Euclidean k-nearest neighbor graphs [image processing examples]. 2004 IEEE International Conference on Acoustics, Speech, and Signal Processing; 2004: IEEE.

40. Yukich JE. Probability theory of classical Euclidean optimization problems: Springer; 2006.

41. Hero AO, Ma B, Michel OJ, Gorman J. Applications of entropic spanning graphs. IEEE signal processing magazine. 2002;19(5):85-95.

42. Costa JA, Hero AO. Geodesic entropic graphs for dimension and entropy estimation in manifold learning. IEEE Transactions on Signal Processing. 2004;52(8):2210-21.

43. Narayan A, Berger B, Cho H. Assessing single-cell transcriptomic variability through density-preserving data visualization. Nature Biotechnology. 2021:1-10.
