# Supplementary material for: MIAAIM: Multi-omics image integration with dimensional reduction for tissue state mapping
Source: PLoS Comput Biol. 2026 May 26;22(5):e1014274. doi: 10.1371/journal.pcbi.1014274 (PMC13225665; doi:10.1371/journal.pcbi.1014274)
Supplement: S4 Note — (DOCX) [file pcbi.1014274.s017.docx]

**S4 Note**

**Estimating pixel embedding dimensionality**

Given a fixed $d$-dimensional image $I_{F}: \mathbb{R}^{d}\to\mathbb{R}^{2}$ with domain $\Omega_{F}$ and a moving $q$-dimensional image $I_{M}: \mathbb{R}^{q}\to\mathbb{R}^{2}$ with domain $\Omega_{m}$, we aim to optimize

$$\begin{aligned} \hat{\mu}=\underset{\mu}{arg min} S (T_{\mu};I_{F}, I_{M}, \Omega_{F}),\#\left( 1 \right) \end{aligned}$$

where $T_{\mu}: \Omega_{F}\to\Omega_{M}$ is a smooth transformation defined by the vector of parameters $\mu\subset\mathbb{R}^{M}$, and $S$ is a similarity measure maximized when $I_{M}{\circ T}_{\mu}$ and $I_{F}$ are aligned.

**Differential geometry and manifold learning:** MIAAIM’s manifold alignment scheme uses the entropic graph-based Rényi $\alpha$-mutual information ($\alpha$-MI) (24) as the similarity measure $S$ in **Equation 1**, which extends to manifold representations of images (i.e., compressed images) embedded in Euclidean space with potentially differing dimensionalities. This measure is justified in the HDIreg manifold alignment scheme through a notion of *intrinsic information (*i.e., entropy) of a manifold. Here, we introduce basic differential geometric concepts to apply existing foundations of intrinsic manifold entropy estimation with the UMAP algorithm. We assume familiarity with the definition of a topological space, smooth manifolds, and Riemannian manifolds. Manifolds are assumed to be smooth and closed – that is, compact and without boundary – or diffeomorphic to closed $n$-dimensional discs in $\mathbb{R}^{n}$.

**Terminology overview.** A Riemannian metric $g$ associates to each point $y\boldsymbol{\in}\mathcal{M}$ a smoothly varying inner product $g_{\boldsymbol{y}} (\cdot, \cdot)$ acting on vectors tangent to $\mathcal{M}$ at $y$. A Riemannian manifold ($\mathcal{M,}g)$ is a smooth manifold $\mathcal{M}$ with a Riemannian metric $g$. The metric induces a Riemannian volume element ${}_{g}$ written in terms of the metric $g$ and local coordinates $x={(x}_{1},\ldots,x_{n})$ as ${}_{g}=\sqrt{\det g (x)}dx_{1}{\wedge\ldots\wedge dx}_{n}$ where $\wedge$ is the wedge product. This volume form defines a measure ${}_{g}$ on $\mathcal{M}$, where the volume of $\mathcal{M}$ is given by $\mathrm{Vol}\left( \mathcal{M} \right)=\int_{\mathcal{M}} {}_{g}(dx)$. Integration of a ${}_{g}$-measurable function $f$ is similarly written $\int_{\mathcal{M}} f(x){}_{g}$ or $\int_{\mathcal{M}} f(x){}_{g}(dx)$. An immersion of a smooth$n$-manifold $\mathcal{M}$ into $\mathcal{N}$ is a differentiable mapping $\psi\mathcal{:M\to N}$ such that$d\psi_{p}:T_{p}\mathcal{M\to}T_{\psi(p)}\mathcal{N}$ is injective for all points $p\mathcal{\in M}$. $\psi$ is therefore an immersion if its derivative is injective everywhere. An embedding between smooth manifolds $\mathcal{M}$ and $\mathcal{N}$ is a smooth function $\mathcal{:M\to N}$ that is an immersion and an embedding of topological spaces (i.e., it is an injective homeomorphism).

Let ($\mathcal{M,}g)$ be a compact, $n$-dimensional Riemannian manifold with metric $g$ immersed in an ambient $\mathbb{R}^{d},$ where $n\ll d$, and let $\mathcal{X}_{j}=\{X_{1},\ldots,X_{j}\}$ be a set of independent and identically distributed random vectors with values drawn from a distribution supported on $\mathcal{M}$. Define $\mathcal{U}_{j}=\{U_{1},\ldots,U_{j}\}$ to be a finite collection of open neighborhoods of each $\mathcal{X}_{j}$. A relaxed, general view of manifold learning is that it aims find an embedding $\hat{}$ within a family $\mathcal{F}$ that minimizes a measure of distortion $D$ between $\mathcal{U}_{j}$ and $\mathcal{(U}_{j})=\{\left( U_{1} \right),...,\left( U_{j} \right)\}$. In this view, the manifold learning problem can expressed as

$$\begin{aligned} \hat{}=\underset{\mathcal{\in F}}{arg min} D (\mathcal{U}_{j}, (\mathcal{U}_{j})),\#\left( 2 \right) \end{aligned}$$

where $\mathcal{F}$ represents a family of embeddings $U_{i}\mapsto\left( U_{i} \right), i=1,\ldots,j$. In machine learning settings, open neighborhoods $U_{i}\in\mathcal{U}_{j}$ of $X_{i}\in\mathcal{X}_{j}$ are often defined as KNN neighborhoods with distances (or probabilistic encodings thereof (37)) in each neighborhood approximated with a positive definite kernel (38), which allows the computation of inner products in a Riemannian framework. More strictly, the metric in these local neighborhoods is often assumed to be Euclidean, which is termed a local isometry assumption. Distortion measures vary by algorithm, with some emphasizing global compatibility between neighborhoods and others emphasizing local structure (for examples, see **S3 Note, HDIprep dimension reduction validation**). We are interested in the volume of these open neighborhoods, pairwise distances of points within them, and their distortion in embedding spaces as dimensionality scales, as these components provide a way to quantify the *intrinsic Rényi* $\alpha$*-entropy*.

**Entropic graph estimators.** Given a Lebesgue density $f$ and identically distributed random vectors $X_{1},\ldots,X_{n}$ with values in a compact subset of $\mathbb{R}^{d}$, the *extrinsic* Rényi $\alpha$-entropy of $f$ is given by:

$$\begin{aligned} H_{\alpha}^{\mathbb{R}^{d}}\left( f \right)=\frac{1}{1-\alpha}\log\int_{\mathbb{R}^{d}} f^{\alpha}\left( \mathcal{x} \right)d\mathcal{x,}\#\left( 3 \right) \end{aligned}$$

where $\alpha\in(0,1)$.

**Definition 7 (adapted from Costa and Hero (39))**. Let $\mathcal{X}_{n}=\{X_{1},\ldots,X_{n}\}$ be identically distributed random vectors with values in a compact subset of $\mathbb{R}^{d}$, the nearest neighbor of $X_{i}\in\mathcal{X}_{n}$ under the Euclidean metric is given by:

$$\begin{aligned} \underset{X\boldsymbol{\in}\mathcal{X}_{n}\backslash\{ X_{i}\}}{arg min} \left\| {X-X}_{i} \right\|_{2}.\#\left( 4 \right) \end{aligned}$$

A $k$-nearest neighbor (KNN) graph puts and edge between each $X_{i}\in\mathcal{X}_{n}$ and its $k$-nearest neighbors. Let$\Gamma_{k,i}=\Gamma_{k,i} (\mathcal{X}_{n})$ be the set of $k$-nearest neighbors of $X_{i}\in\mathcal{X}_{n}$. Then the total edge length of the KNN graph for $\mathcal{X}_{n}$ is given by:

$$\begin{aligned} L_{\gamma,k}\left( \mathcal{X}_{n} \right)=\sum_{i=1}^{n} \sum_{X\in\Gamma_{k,i}} \left\| {X-X}_{i} \right\|_{2}^{\gamma},\#\left( 5 \right) \end{aligned}$$

where $\gamma>0$ is a power-weighting constant.

In practice, the *extrinsic* Rényi $\alpha$-entropy of $f$ can be suitably approximated using a class of graphs known as continuous quasi-additive graphs, including k-nearest neighbor (KNN) Euclidean graphs (40), as their edge lengths asymptotically converge to the Rényi $\alpha$-entropy of feature distributions as the number of feature vectors increases (41). This property leads to the convergence of KNN Euclidean edge lengths to the *extrinsic* Rényi $\alpha$-entropy of a set of random vectors with values in a compact subset of $\mathbb{R}^{d}$ where $d\geq2$ (39). This is a direct corollary of the **Beardwood-Halton-Hammersley Theorem** outlined below.

**Beardwood-Halton-Hammersley (BHH) Theorem (39, 40).** *Let (*$\mathcal{M},g)$ *be a compact Riemannian* $m$*-manifold immersed in an ambient* $\mathbb{R}^{d}$*. Suppose* ${\mathcal{X}_{n}=\{X}_{1},\ldots{,X}_{n}\}$ *are identically distributed random vectors with values in a compact subset of* $\mathbb{R}^{d}$ *and Lebesgue density* $f.$ *Assume* $d\geq2, 1\leq\gamma<d$ *and define* $\alpha=\frac{(d-\gamma)}{d}.$*Then with probability 1,*

$$\begin{aligned} \lim_{n\to\infty} \frac{L_{\gamma,k}\left( \mathcal{X}_{n} \right)}{n^{\frac{d^{'}-\gamma}{d^{'}}}}=\beta_{d,\gamma,k}\int_{\mathcal{M}} f^{\alpha}\left( x \right)dx.\#\left( 6 \right) \end{aligned}$$

The value that determines the right side of the limit in **Equation 6** is the extrinsic Rényi $\alpha$-entropy given by **Equation 3**. When identically distributed random vectors are restricted to a compact smooth $m$-manifold $\mathcal{M}$ immersed in $\mathbb{R}^{d}$, the **BHH Theorem** generalizes to enable an estimation of *intrinsic* Rényi $\alpha$-entropy $H_{\alpha}^{\mathcal{M}}\left( f \right)$ of the multivariate density $f$ on $\mathcal{M}$, defined by

$$\begin{aligned} H_{\alpha}^{\mathcal{M}}\left( f \right)=\frac{1}{1-\alpha}\log\int_{\mathcal{M}} f^{\alpha}\left( y \right)\mu_{g}\left( dy \right),\#\left( 7 \right) \end{aligned}$$

by incorporating the measure $\mu_{g}$ naturally induced by the Riemannian metric via the Riemannian volume element (39). This is formalized by the following theorem by Costa and Hero (39):

**Theorem 1 (Costa and Hero (39))**: *Let (*$\mathcal{M},g)$ *be a compact Riemannian* $m$*-manifold immersed in an ambient* $\mathbb{R}^{d}$*. Suppose* ${\mathcal{Y}_{n}=\{Y}_{1},\ldots{,Y}_{n}\}$ *are identically distributed random vectors of* $\mathcal{M}$ *with bounded density* $f$ *relative to the differential volume element* $\mu_{g}$ *induced by the metric* $g$*. Assume* $d\geq2, 1\leq\gamma<d$ *and define* $\alpha=\frac{(d-\gamma)}{d}.$*Then with probability 1,*

$$\begin{aligned} \lim_{n\to\infty} \frac{L_{\gamma,k}\left( \mathcal{Y}_{n} \right)}{n^{\frac{d^{'}-\gamma}{d^{'}}}}=\left\{ \begin{aligned} \begin{aligned} \infty, d^{'}<m \\ \beta_{m,\gamma,k}\int_{\mathcal{M}} f^{\alpha}\left( y \right)\mu_{g}\left( dy \right), d^{'}=m \\ \end{aligned} \\ 0,d^{'}>m , \end{aligned} \right.\#\left( 8 \right) \end{aligned}$$

*where* $\beta_{m,\gamma,k}$ *is a constant that is independent of* $f$ *and (*$\mathcal{M},g)$*. Similarly, the expectation* $E\left[ L_{\gamma,k}\left( \mathcal{Y}_{n} \right) \right]/n^{\alpha}$ *converges to the same limit.*

The quantity that determines the limit when $d^{'}=m$ is the intrinsic Renyi alpha entropy of $f$ given by **Equation 7. Theorem 1** has been used in conjunction with manifold learning algorithms Isomap and a variant C-Isomap to estimate the intrinsic dimensionality of embedded manifolds (42). In contrast to these results that use all pairwise geodesic approximations for each point in the data set to estimate the $\alpha$-entropy, we aim to provide a similar formulation using local neighborhoods alone, following the results of our dimension reduction benchmark, which shows that local information preserving algorithms are well-suited for the task of high-dimensional image data compression (**S1-3 Figs**).

**Entropic graph estimators and local information of embedded manifolds:** Two concepts are used to motivate an estimate of the intrinsic information of multivariate probability distributions supported by embedded manifolds in Euclidean space using the UMAP algorithm and the **BHH Theorem**: (i) the compactness of embedded manifolds and (ii) the conservation of local distances (KNN graph lengths) and volumes in idealized scenarios. We address (i) with a simple proof, and we provide a motivational example to address (ii).

**Proposition 1.** *Let* $n>d$ *and suppose that* $\mathcal{M}$ *is a compact manifold of dimension* $r$ *with* $r\leq d$ *that is immersed in ambient* $\mathbb{R}^{n}$*. Then the image* $(\mathcal{M})$ *of* $\mathcal{M}$ *under an embedding* $:\mathcal{M\to}\mathbb{R}^{d}$ *is compact.*

*Proof.* Let ($\mathcal{M,}g)$ be a compact Riemannian manifold (e.g., a manifold constructed with UMAP) with metric $g$ in an ambient $\mathbb{R}^{n}$ and an embedding of $\mathcal{M}$ in $\mathbb{R}^{d}$. Since is an embedding, it is continuous and therefore takes compact sets to compact sets. $∎$

**Proposition 1** shows that a $d$-dimensional Euclidean embedding of a compact Riemannian manifold takes values in a compact subset of $\mathbb{R}^{d}$, a necessary condition of the **BHH Theorem**. That is an embedding not strictly needed (it only needs to be continuous). To extend the **BHH Theorem** to the calculation of intrinsic$\alpha$-entropy as in **Equation 7**, the volume elements induced via embedding need to be well approximated. This applies to any dimension reduction algorithm that can provably preserve distances at the image of embedded neighborhoods of a compact manifold in Euclidean space. We do not provide a proof that UMAP preserves distances within open neighborhoods of data points, though this would be ideal. Instead, we assume that this ideal scenario exists, and we describe how to estimate an “optimal” dimensionality for projecting data to satisfy this assumption.

In contrast to global data preserving algorithms – such as Isomap – that calculate all pairwise geodesic distances or approximations thereof with landmark based approaches, UMAP approximates geodesic distances in open neighborhoods local to each point under a local isometry assumption. Given identically distributed random vectors $Y_{1},\ldots,Y_{n}$ with values constrained to lie on a compact Riemannian manifold $\mathcal{M}$, geodesics between samples $Y_{i}$ and $Y_{j}$ in a local neighborhood are encoded with UMAP using a scaled exponential distribution(43):

$$\begin{aligned} P_{j|i}=\exp\left( \frac{-\left\| Y_{i}-Y_{j} \right\|-\rho_{i}}{\sigma_{i}} \right),\#\left( 9 \right) \end{aligned}$$

$$\begin{aligned} P_{ij}=P_{j|i}+P_{i|j}-P_{j|i}P_{i|j},\#\left( 10 \right) \end{aligned}$$

where $\rho_{i}$ is the distance from vector $Y_{i}$ to its nearest neighbor and $\sigma_{i}$ is an adaptively chosen normalization factor. Using the terminology in **Equation 2,** the objective of embedding in UMAP is given by minimizing the fuzzy simplicial set cross-entropy (**Definition 1**), which represents distortion $D$. Given a distribution $P_{ij}$ encoding geodesics between samples $Y_{i}$ and $Y_{j}$ and probability distribution $Q_{ij}$ encoding distances between samples ${(Y}_{i}$) and $(Y_{j})$, the cross-entropy loss is (adapted from Narayan et al. (43)):

$$\begin{aligned} CE\left( \left. P \right\|Q \right)=-\sum_{ij} P_{ij}\log Q_{ij}+ (1-P_{ij})log (1-Q_{ij}),\#\left( 11 \right) \end{aligned}$$

where $Q_{ij}$ is the probability distribution formed from low dimensional positions of embedded vectors ${(Y}_{i}$) and $(Y_{j})$ by $Q_{ij} (a,b)=\frac{1}{\left( 1+a\left\| {(Y}_{i})-(Y_{j}) \right\|_{2}^{2b} \right)}$ with $a,b$ user-defined parameters to control embedding spread.

Minimizing **Equation 11** is not, in general, a convex optimization problem. For a fixed embedding dimensionality, optimization over the family $\mathcal{F}$ from **Equation 2** is restricted to a subset that represents, in the best case, a local optimum in the sense that higher dimensionalities are likely to reduce error. We include a larger family of functions to more accurately approach an optimal embedding of geodesic distances within open neighborhoods of each point through the estimation of embedding dimensionalities, as outlined in the HDIprep workflow in a “pseudo-global” optimization procedure.

To estimate embeddings that minimize distortion of local distances and volumes, we view increases in the dimensionality of real-valued data by viewing increases in dimensionality as exponential increases in potential positions of points (i.e., increasing copies of the real line, $\mathbb{R}^{n}$). The relationship between radii of open neighborhoods, volume, and density of manifolds in a learning setting has been considered by Narayan et al. (43) in an application where density is preserved with a given dimensionality for embeddings by altering open neighborhood radii; however, we can extend this in a simpler scenario of volume preservation under fixed radii to infer a relationship between dimensionality and distances within neighborhoods in Euclidean space. Consider the following example:

Let $\mathcal{M}$ be an immersed in an ambient $\mathbb{R}^{d}$, let $Y_{i}\in\mathcal{M}$. Suppose that the $k$-nearest neighbors of $Y_{i}$ are uniformly distributed in a ball $B_{r_{d}}$ of radius $r_{d}$. Under the local isometry assumption (i.e., locally Euclidean metric), the volume of the neighborhood scales as $V_{d}\propto r_{d}^{d}$. Assume that an embedding takes the open neighborhood $B_{r_{d}}$ of $Y_{i}$ to an $m$-dimensional ball $B_{r_{m}}\subseteq\mathbb{R}^{d}$ with radius $r_{m}$ while preserving its geometric structure, including the uniform distribution and the pairwise distances between points up to a uniform dilation. The corresponding embedding volume scales as $V_{m}\propto r_{m}^{m}$. Motivated by Narayan et al. (43), if local volume is preserved ($V_{m}=V_{d}$), equating these proportions gives a power law relationship $r_{m}\propto$ $r_{d}^{d/m}$ between the local radii $r_{m}$ in embedding spaces and the original radius of $B_{r_{d}}$ across dimensions.

The local isometry assumption and the condition on the embedding preserving distances up to a uniform dilation imply that metric is uniformly Euclidean (or is a uniformly rescaled Euclidean metric) within $B_{r_{m}}$ and $B_{r_{d}}$. Therefore, geodesic distances within these neighborhoods also scale linearly with neighborhood radius under a uniform dilation. Composing this linear scaling with the cross-dimensional radius scaling $r_{m}\propto$ $r_{d}^{d/m}$ implies that pairwise geodesic distances $\delta_{m}$ and $\delta_{d}$ between points in $B_{r_{m}}$ and $B_{r_{d}}$ also exhibit a power law relationship, $\delta_{m}\propto$ $\delta_{d}^{d/m}$. This power-law relationship provides a heuristic link between embedding dimensionality and the preservation of local geometric structure.

In view of these power-law relationships in neighborhoods constructed by UMAP and their image under an embedding in an idealized case, we can attempt to identify the dimensionality $m$ such that distances within neighborhoods are preserved with an exponential regression modeling the cross-entropy in **Equation 11** with respect to dimensionality. Our experiments indicate that the cross-entropy drops rapidly with only a few dimensions for nearly all samples, and that exponential regression captures this decay well. KNN graph functionals calculated in the estimated optimal embedding space provide the machinery to compute an estimate of the intrinsic $\alpha$-entropy of embedded data manifolds in MIAAIM by applying the **BHH Theorem** across all local neighborhoods as in **Theorem 1**.

The distances outside of open neighborhoods of points are not guaranteed to be accurately modelled in the embedding space with UMAP if one makes the assumptions introduced in our example. Therefore, applying **Theorem 1** in conjunction with UMAP by replacing KNN graph lengths with those obtained with length functionals of geodesic minimal spanning trees (GMST), another type of entropic graph, should not be expected to reproduce the intrinsic entropy originally reported by Costa and Hero (42). Our primary contribution here is combining the KNN intrinsic entropy estimator with a heuristic approach for local information preserving dimension reduction. We wish to extend these results to the setting where two such manifolds are compared to each other, as this serves as the basis of our image registration application. An entropic graph-based estimator of $\alpha$-MI in an image registration setting is described by the following (24):

Let $z\left( x_{i} \right)=[z_{i}\left( x_{i} \right),\ldots,z_{d}\left( x_{i} \right)]$ be a $d$-dimensional vector encoding the features of point $x_{i}$. Let $Z_{f}\left( x \right)=\{z^{f}\left( x_{1} \right),\ldots,z^{f} (x_{N})\}$ be the feature set of a fixed image, $Z_{m}\left( T_{\mu}(x) \right)=\{z^{m} ({T_{\mu}(x}_{1})),\ldots,z^{m} ({T_{\mu}(x}_{N}))\}$ be the feature set of a transformed moving image at points in $T_{\mu}(x)$, and $z_{i}^{fm}=[z^{f}\left( x_{i} \right),z^{m}\left( T_{\mu}\left( x_{i} \right) \right)]$ be the concatenation of the feature vectors of the fixed and transformed moving image at $x_{i}$. Therefore,

$$\begin{aligned} \hat{\alpha-MI}\left( \mu\boldsymbol{;}Z_{f},Z_{m},Z_{fm} \right)=\frac{1}{\alpha-1}\log\frac{1}{N^{\alpha}} \sum_{i=1}^{N} \left( \frac{\Gamma_{i}^{fm}}{\sqrt{\Gamma_{i}^{f}\Gamma_{i}^{m}}\left( \mu\right)} \right)^{2\gamma}\#\left( 12 \right) \end{aligned}$$

is a graph-based estimator for $\alpha$-MI, where $\gamma=d (1-\alpha)$, $0<\alpha<1$, and three graphs

$$\begin{aligned} \Gamma_{i}^{f}=\sum_{i=1}^{k} \left\| z^{f}\left( x_{i} \right)-z^{f}\left( x_{ip} \right) \right\|\#\left( 13 \right) \end{aligned}$$

$$\begin{aligned} \Gamma_{i}^{m}\left( \mu\right)=\sum_{i=1}^{k} \left\| z^{m} ({T_{\mu} (x}_{i}))-z^{m} (T_{\mu} (x_{ip})) \right\|\#\left( 14 \right) \end{aligned}$$

$$\begin{aligned} \Gamma_{i}^{fm}\left( \mu\right)=\sum_{i=1}^{k} \left\| z^{fm} ({{x_{i},T}_{\mu}(x}_{i}))-z^{fm} ({x_{ip},T}_{\mu}(x_{ip})) \right\|\#\left( 15 \right) \end{aligned}$$

represent the Euclidean graph functionals (lengths) of a feature vector $z$ to its $p^{th}$ nearest neighbor over $k$ considered nearest neighbors.

Ultimately, Rényi $\alpha$-MI provides a quantitative measure of association between the intrinsic structure of multiple embeddings. The Rényi $\alpha$-MI measure extends to feature spaces of arbitrary dimensionality, which MIAAIM utilizes in combination with its image compression method to quantify similarity between embeddings of image pixels in potentially differing dimensionalities.

**Supporting References**

1. McDonnell LA, Heeren RM. Imaging mass spectrometry. Mass spectrometry reviews. 2007;26(4):606-43.

2. Giesen C, Wang HA, Schapiro D, Zivanovic N, Jacobs A, Hattendorf B, et al. Highly multiplexed imaging of tumor tissues with subcellular resolution by mass cytometry. Nature methods. 2014;11(4):417-22.

3. Angelo M, Bendall SC, Finck R, Hale MB, Hitzman C, Borowsky AD, et al. Multiplexed ion beam imaging of human breast tumors. Nature medicine. 2014;20(4):436.

4. <https://github.com/ionpath/mibilib>.

5. Goltsev Y, Samusik N, Kennedy-Darling J, Bhate S, Hale M, Vazquez G, et al. Deep profiling of mouse splenic architecture with CODEX multiplexed imaging. Cell. 2018;174(4):968-81. e15.

6. Lin J-R, Izar B, Wang S, Yapp C, Mei S, Shah PM, et al. Highly multiplexed immunofluorescence imaging of human tissues and tumors using t-CyCIF and conventional optical microscopes. Elife. 2018;7.

7. Rashid R, Gaglia G, Chen Y-A, Lin J-R, Du Z, Maliga Z, et al. Highly multiplexed immunofluorescence images and single-cell data of immune markers in tonsil and lung cancer. Scientific data. 2019;6(1):1-10.

8. Gut G, Herrmann MD, Pelkmans L. Multiplexed protein maps link subcellular organization to cellular states. Science. 2018;361(6401).

9. Rodriques SG, Stickels RR, Goeva A, Martin CA, Murray E, Vanderburg CR, et al. Slide-seq: A scalable technology for measuring genome-wide expression at high spatial resolution. Science. 2019;363(6434):1463-7.

10. Abdelmoula WM, Skraskova K, Balluff B, Carreira RJ, Tolner EA, Lelieveldt BP, et al. Automatic generic registration of mass spectrometry imaging data to histology using nonlinear stochastic embedding. Anal Chem. 2014;86(18):9204-11.

11. Abdelmoula WM, Regan MS, Lopez BGC, Randall EC, Lawler S, Mladek AC, et al. Automatic 3D Nonlinear Registration of Mass Spectrometry Imaging and Magnetic Resonance Imaging Data. Anal Chem. 2019;91(9):6206-16.

12. Li L, Shiradkar R, Gottlieb N, Buzzy C, Hiremath A, Viswanathan VS, et al. Multi-scale statistical deformation based co-registration of prostate MRI and post-surgical whole mount histopathology. Med Phys. 2024;51(4):2549-62.

13. Huizinga W, Poot DH, Guyader JM, Klaassen R, Coolen BF, van Kranenburg M, et al. PCA-based groupwise image registration for quantitative MRI. Med Image Anal. 2016;29:65-78.

14. Guyader JM, Huizinga W, Fortunati V, Poot DHJ, Veenland JF, Paulides MM, et al. Groupwise Multichannel Image Registration. IEEE J Biomed Health Inform. 2019;23(3):1171-80.

15. Klein S, Staring M, Murphy K, Viergever MA, Pluim JP. elastix: a toolbox for intensity-based medical image registration. IEEE Trans Med Imaging. 2010;29(1):196-205.

16. Mahapatra D, Antony B, Sedai S, Garnavi R, editors. Deformable medical image registration using generative adversarial networks. 2018 IEEE 15th International Symposium on Biomedical Imaging (ISBI 2018); 2018 4-7 April 2018.

17. Sorzano CO, Thevenaz P, Unser M. Elastic registration of biological images using vector-spline regularization. IEEE Trans Biomed Eng. 2005;52(4):652-63.

18. Schapiro D, Sokolov A, Yapp C, Muhlich JL, Hess J, Lin J-R, et al. MCMICRO: A scalable, modular image-processing pipeline for multiplexed tissue imaging. bioRxiv. 2021.

19. Schapiro D, Jackson HW, Raghuraman S, Fischer JR, Zanotelli VR, Schulz D, et al. histoCAT: analysis of cell phenotypes and interactions in multiplex image cytometry data. Nature methods. 2017;14(9):873.

20. Berg S, Kutra D, Kroeger T, Straehle CN, Kausler BX, Haubold C, et al. ilastik: Interactive machine learning for (bio) image analysis. Nature Methods. 2019:1-7.

21. Schindelin J, Arganda-Carreras I, Frise E, Kaynig V, Longair M, Pietzsch T, et al. Fiji: an open-source platform for biological-image analysis. Nature methods. 2012;9(7):676-82.

22. Bankhead P, Loughrey MB, Fernández JA, Dombrowski Y, McArt DG, Dunne PD, et al. QuPath: Open source software for digital pathology image analysis. Scientific reports. 2017;7(1):1-7.

23. Sofroniew N, Talley Lambert, Evans, K., Nunez-Iglesias, J., Yamauchi, K., Solak, A. C., Buckley, G., Bokota, G., Tung, T., Ziyangczi, Freeman, J., Boone, P., Winston, P., Loic Royer, Har-Gil, H., Axelrod, S., Rokem, A., Bryant, Hector, Mars Huang, Pranathi Vemuri, Dunham, R., Jakirkham, Siqueira, A. D., Bhavya Chopra, Wood, C., Gohlke, C., Bennett, D., DragaDoncila & Perlman, E. napari/napari: 0.3.5. (Zenodo, 2020). 2020.

24. Staring M, Van Der Heide UA, Klein S, Viergever MA, Pluim JP. Registration of cervical MRI using multifeature mutual information. IEEE transactions on medical imaging. 2009;28(9):1412-21.

25. Maaten Lvd, Hinton G. Visualizing data using t-SNE. Journal of machine learning research. 2008;9(Nov):2579-605.

26. McInnes L, Healy J, Melville J. Umap: Uniform manifold approximation and projection for dimension reduction. arXiv preprint arXiv:180203426. 2018.

27. Moon KR, van Dijk D, Wang Z, Gigante S, Burkhardt DB, Chen WS, et al. Visualizing structure and transitions in high-dimensional biological data. Nature Biotechnology. 2019;37(12):1482-92.

28. Tenenbaum JB, De Silva V, Langford JC. A global geometric framework for nonlinear dimensionality reduction. science. 2000;290(5500):2319-23.

29. Kim H, Park H. Sparse non-negative matrix factorizations via alternating non-negativity-constrained least squares for microarray data analysis. Bioinformatics. 2007;23(12):1495-502.

30. Jolliffe IT, Cadima J. Principal component analysis: a review and recent developments. Philosophical Transactions of the Royal Society A: Mathematical, Physical and Engineering Sciences. 2016;374(2065):20150202.

31. Leland M, John H, Nathaniel S, Lukas G. UMAP: Uniform Manifold Approximation and Projection. Journal of Open Source Software. 2018;3(29):861.

32. Connor Meehan SM, and Wayne Moore. Uniform Manifold Approximation and Projection (UMAP). <https://www.mathworks.com/matlabcentral/fileexchange/71902>, MATLAB Central File Exchange.2020.

33. Ulyanov D. Multicore-tsne. GitHub Repos GitHub. 2016.

34. Pedregosa F, Varoquaux G, Gramfort A, Michel V, Thirion B, Grisel O, et al. Scikit-learn: Machine learning in Python. Journal of machine learning research. 2011;12(Oct):2825-30.

35. Rueckert D, Sonoda LI, Hayes C, Hill DL, Leach MO, Hawkes DJ. Nonrigid registration using free-form deformations: application to breast MR images. IEEE transactions on medical imaging. 1999;18(8):712-21.

36. Lowekamp BC, Chen DT, Ibáñez L, Blezek D. The design of SimpleITK. Frontiers in neuroinformatics. 2013;7:45.

37. Sun K, Marchand-Maillet S, editors. An information geometry of statistical manifold learning. International Conference on Machine Learning; 2014: PMLR.

38. Jayasumana S, Hartley R, Salzmann M, Li H, Harandi M. Kernel methods on Riemannian manifolds with Gaussian RBF kernels. IEEE transactions on pattern analysis and machine intelligence. 2015;37(12):2464-77.

39. Costa JA, Hero AO, editors. Manifold learning using Euclidean k-nearest neighbor graphs [image processing examples]. 2004 IEEE International Conference on Acoustics, Speech, and Signal Processing; 2004: IEEE.

40. Yukich JE. Probability theory of classical Euclidean optimization problems: Springer; 2006.

41. Hero AO, Ma B, Michel OJ, Gorman J. Applications of entropic spanning graphs. IEEE signal processing magazine. 2002;19(5):85-95.

42. Costa JA, Hero AO. Geodesic entropic graphs for dimension and entropy estimation in manifold learning. IEEE Transactions on Signal Processing. 2004;52(8):2210-21.

43. Narayan A, Berger B, Cho H. Assessing single-cell transcriptomic variability through density-preserving data visualization. Nature Biotechnology. 2021:1-10.
