# Supplementary material for: MIAAIM: Multi-omics image integration with dimensional reduction for tissue state mapping
Source: PLoS Comput Biol. 2026 May 26;22(5):e1014274. doi: 10.1371/journal.pcbi.1014274 (PMC13225665; doi:10.1371/journal.pcbi.1014274)
Supplement: Attachment — Submitted filename: 2026-02_MIAAIM_Pointbypoint.docx [file pcbi.1014274.s019.docx]

Below please find our point-by-point responses detailing how we have endeavored to directly and clearly address each comment or item. We thank the reviewers for their helpful critiques and are grateful for the improvements to the manuscript resulting from addressing each point raised.

Reviewer #1: The classification of prostate cancer Gleason scores is a powerful application of the MIAAIM framework. The manuscript already contains the critical data showing that the integrated multimodal model outperforms models based on single modalities. This result is a cornerstone of the paper's argument, as it provides direct evidence that multimodal integration yields a more complete and predictive representation of tissue states.

To make this method more impactful, it is suggested add an experiment to the presentation of these results: consider adding a summary table or a simple bar chart in the main text that directly compares the final classification accuracy (e.g., class-balanced accuracy) for models trained on:

1. IMC features only (including single-cell and neighborhood interaction scores).

2. MSI features only (including single-cell and neighborhood interaction scores).

3. The fully integrated IMC + MSI model.

While this information is mentioned in the text, a dedicated figure or table would provide a clear visualization of the performance gain achieved through multimodal integration.

We thank the reviewer for their thoughtful comment. In agreement with presenting the information more clearly and concisely, we have added Supplementary Table 3 (included below). The table provides the baseline accuracy of a logistic regression model classifying the 3 prostate cancer grades. As described in the manuscript, using both MSI and IMC modalities results in the most accurate model at 65±0.07% accuracy. The model based on the IMC data alone is 58±0.02% accurate, while the MSI only model is 55±0.14%. Though IMC alone outperforms MSI, the MSI data adds information to the model since the combined IMC + MSI model outperforms either data modality individually.

| **Data Modality** | **Accuracy±S.D.** | **Improvement Over Random** |
| --- | --- | --- |
| MSI+MSI interaction + IMC + IMC interaction | 0.6498±0.0738 | 31.65% |
| IMC+IMC interaction | 0.5791±0.0239 | 24.58% |
| IMC Interaction | 0.5694±0.0246 | 23.60% |
| IMC | 0.5574±0.0263 | 22.40% |
| MSI+MSI interaction | 0.5506±0.1448 | 21.72% |
| MSI Interaction | 0.5279±0.1283 | 19.45% |
| MSI | 0.4967±0.0572 | 16.33% |

Reviewer #2: Summary:

This paper describes MIAAIM, an open-source and modular computational framework for aligning and integrating multimodal, high-dimensional tissue imaging. By combining concepts such as UMAP and entropic-graph-based mutual information, MIAAIM can align data from disparate modalities. The authors focus on aligning data from imaging mass cytometry (IMC), mass spectrometry imaging (MSI), and histology. They apply the framework to a large diabetic foot ulcer (DFU) biopsy and to multi-core prostate cancer tissue microarrays (TMAs), demonstrating MIAAIM’s ability to extract biologically meaningful multimodal single-cell and spatial features.

Major Comments:

My main concern is the lack of quantitative benchmarking. The α–mutual information alignment method is conceptually elegant, but registration quality is reported primarily through qualitative visual inspection, which is a serious limitation. Commonly used quantitative metrics such as Dice coefficient, target registration error (TRE), or mutual information gain relative to baseline methods (e.g., Elastix) should be reported. I cannot foresee a path toward publication without explicit comparisons. These results are critical to clarify MIAAIM’s robustness and, more importantly, justify its novelty relative to existing registration tools. Lastly, a brief discussion of false discovery rates or validation strategies would strengthen the biological claims.

We thank the reviewer for this suggestion. In order to clarify the benefit our approach offers compared to conventional methods, we have added a new experiment to compare MIAAIM’s registration framework to the conventional landmark-based approach with Elastix **(Supplementary Fig. 9)**. This approach is standard for aligning multimodal images in the context of multimodal bioimaging (e.g., it is used in SCiLS Lab, a widely used MSI data analysis software, and is the dominant approach for nonlinear registration in Image J).

The experiment quantifies the mutual information gain that MIAAIM provides as compared to the Elastix framework between the IMC and MSI modalities. Our results indicate that alignment with MIAAIM improves the alignment quality for approximately 50% of samples in the prostate cancer TMA dataset, and does not reduce alignment quality in the other half. As noted in the manuscript (Line 223), for the 14 of 30 samples that MIAAIM improves alignment, MIAAIM provided a quantifiable 70-95% gain in 10 samples and a ~16-70% gain in 4 samples. Inspection of samples that benefit from registration with MIAAIM indicate that using the α–mutual information within the MIAAIM framework corrects for subtle misalignments (<20 $\mu$m) that the alternative approach does not capture, and which are critical at single-cell resolutions **(Supplementary Fig. 9b).**

We similarly appreciate the suggestion to provide a brief discussion of FDR and validation strategies to improve confidence in biological inferences. We include an expanded portion in the discussion to directly address both strategies for validation as well as the implementation of statistical procedures (Lines 445-469).

Minor Comments:

• Standardize reference formatting (several in-text citations have unmatched parentheses).

We thank the reviewer for this point, citation formatting is now corrected.

• Ensure all acronyms are defined at first mention.

We have reviewed the manuscript to ensure that acronyms are defined at first mention. In some cases, we reiterate for clarity (e.g. DFU). In other instances, such as CD3 (cluster of differentiation 3) or t-SNE, we have not included definitions for those acronyms given their common usage though have provided the necessary contextual information. However, if the reviewer or editor indicates that definitions within the text for these are preferred, we are glad to include them. Alternatively, we can provide a supplementary table that lists all acronyms and their definitions.

• Clarify how manual landmarks were used in DFU alignment and whether this step introduces bias.

We thank the reviewer for this suggestion and have revised the text within the “Multimodal image registration” section to clarify landmark utilization in the DFU alignment, as well as PC TMA cores, and the impact on imparting bias (Line 739-768).

Additional changes:

Small typographical errors appearing in the original submission have been fixed. In addition, light edits have been made to **Supplementary Note 4** to enhance the exposition and clarity of the approach to estimate the dimensionality of pixel embeddings using UMAP. Definitions that are standard in the differential geometry literature have been consolidated to a “terminology overview” section, rather than being listed one-by-one. Ambiguities and redundant assumptions in the motivating example have also been tightened or otherwise removed. These edits do not affect any of the results, methods, or conclusions drawn in the paper.
